# Early prediction of wind turbine anomalies using 1D-CNN and temporal feature engineering on multi-source SCADA data

**DOI:** 10.1038/s41598-026-41571-7

**Published:** 2026-03-21

**Authors:** Mohamed Maher Ata, Shorok Osama, Mai Ramadan Ibraheem, Ahmed R. Abas

**Affiliations:** 1 School of Computational Sciences and Artificial Intelligence (CSAI), Zewail City of Science and Technology, October Gardens, 6th of October City, 12578 Giza Egypt; 2 Department of Computer Science, Faculty of Computer and Informatics, Zagazig University, Zagazig, 44519 Egypt; 3 Department of Information Technology (IT), Faculty of Computers and Information, Kafrelsheikh University, Kafrelsheikh, 33516 Egypt

**Keywords:** Wind turbines, Deep learning, CNN, TCN, RNN, LSTM, Bi-LSTM, GRU predictive maintenance, Renewable Energy, Hybrid model, Attention, Energy science and technology, Engineering, Mathematics and computing

## Abstract

Early and accurate detection of anomalies in wind turbines is critical for ensuring system reliability, minimizing unplanned downtime, and reducing maintenance costs in large-scale renewable energy infrastructures. In this study, we propose a robust deep learning framework for wind turbine anomaly detection, leveraging a newly constructed dataset that integrates Supervisory Control and Data Acquisition (SCADA) data from three distinct wind farms. Extensive preprocessing and domain-specific temporal feature engineering were employed to capture complex patterns and enhance model generalizability across heterogeneous data sources. A comparative evaluation of several state-of-the-art deep learning models—including 1D Convolutional Neural Networks (1D-CNN), Recurrent Neural Networks (RNN), Long Short-Term Memory (LSTM), Bidirectional LSTM (Bi-LSTM), and Gated Recurrent Units (GRU); was conducted using standard classification metrics. Among these, the 1D-CNN consistently outperformed the recurrent models, achieving an accuracy and F1-score of 85%. This performance is attributed to the model’s capacity to effectively learn localized temporal dynamics in multivariate time series data. The findings demonstrate that a carefully designed 1D-CNN architecture, combined with strategic temporal feature engineering and multi-source data fusion, offers a scalable and accurate solution for early fault detection in wind turbine systems. This work lays the foundation for intelligent condition monitoring systems in the renewable energy sector. We then propose and evaluate a hybrid CNN-LSTM architecture augmented with an attention mechanism. This model leverages both CNN’s strength of extracting local features. And LSTM capacity to capture temporal dependencies, while the attention layer dynamically focuses on the most important segments of the sequence. Our findings show that the suggested hybrid model performs noticeably better than the independent base models, attaining higher generalization and accuracy. This work advances wind turbine fault detection through creating a diverse, multi-source wind farm dataset for superior generalizability; pioneering a reproducible benchmarking framework across deep learning models on heterogeneous data; and a hybrid CNN-LSTM with attention, surpassing baselines by 2% while enabling practical decision-making.

## Introduction

While the world is transitioning to sustainable sources of energy, renewable sources of energy are slowly taking over fossil fuels as the basis of power generation in the future. Among these, wind energy stands out as one of the fastest growing and most promising sectors, largely due to its safety, reliability, and scalability. However, like all complex machinery, they are not perfect, gearboxes wear out, bearings fail, blades crack, and electrical systems fail. When these failures happen, their repair is expensive, and the downtime equals lost energy production. Their operation and maintenance bring significant challenges. Exposed to harsh weather conditions, wind turbines are most susceptible to damage, and their astronomical replacement prices—are evidence of the importance of effective maintenance measures.

In recent years, wind turbines have rapidly grown in numbers, scale and complexity. And it is expected to increase by 50% to over 30 GW by 2027^1^. Proper maintenance for wind turbines would reduce downtime, failures and increase efficiency. The monetary loss of wind turbine failure is considerable. Studies have shown that offshore wind turbines undergo ten faults each year on average, the majority of which involve minor repairs, but a significant proportion of which require costly major repairs or replacement making proactive maintenance very valuable^2^. Critical systems such as pitch/hydraulic systems, generators, and converters are most prone to failure, the offshore turbines experiencing higher failure rates compared to onshore turbines. In an attempt to mitigate such risks, asset owners are relying more on advanced monitoring techniques, employing SCADA systems and tailored sensors to enable proactive maintenance approaches.

This is where artificial intelligence (AI) and deep learning come in. Over the past decade, AI has revolutionized everything from medical diagnostics to self-driving cars, and now, it’s transforming wind turbine maintenance. Traditional fault detection methods ranging from physical modeling to signal processing are now complemented by data driven approaches leveraging the vast amount of data collected from operating wind turbines^3^. Deep learning is a branch of machine learning that excels at modeling complex and non-linear relationships in large datasets. It has shown promising results in identifying subtle patterns indicating faults in wind turbines^4,5^. These AI systems can detect early signs of unusual vibrations, or electrical issues, allowing engineers to fix problems before they lead to costly breakdowns. However, it still faces challenges such as data quality, lack of publicly available datasets, and model generalization^6^. Proper feature selection and robust methodology that can generalize across different wind turbines and operational conditions are critical to ensure appropriate maintenance^7^. The benefits are huge; they result in fewer sudden and unexpected failures as AI can give advanced warnings. Fixing small issues early lowers the maintenance cost and is cheaper than replacing entire components after a failure. Results in more efficient energy production as less downtime means more consistent power generation.

The main goal of this study is to create a uniform benchmark for the following widely used deep learning architectures in a single, shared experimental environment: 1D CNN, TCN, RNN, LSTM, Bi-LSTM, and GRU. While a number of recent studies have demonstrated improved performance with enhancements to these architectures, e.g. with attention, hybrid CNN–RNN architectures, or using residual connections^8,9^, we have the primary aim of providing a fair and reproducible comparison of the standard baseline models. We believe it is necessary to have a common baseline that can later serve as a reference by which more advanced or domain-specific changes can be evaluated as part of future studies.

expanding on this, we also examine how well a hybrid CNN-LSTM model enhanced with an attention mechanism performs. This second experiment shows how performance can be further improved beyond the defined baselines through architectural improvements.

The main contributions of this paper can be summarized as follows:

- Integrating several datasets sourced from different places to build a more robust and diverse dataset, enhancing the generalizability and reliability of the built models.
- Proposing a benchmarking framework by systematically evaluating multiple deep learning architectures across heterogeneous wind farm datasets. Providing a reproducible baseline for future studies that aim to build upon or extend it. To our knowledge, this type of cross-dataset benchmarking for wind turbine fault detection has not been comprehensively addressed in prior work.
- Proposing a hybrid CNN-LSTM model with an attention mechanism. This architecture combines the convolutional and recurrent layers’ strengths, and the attention mechanism enables the model to dynamically focus on the sequence’s most important features, improving performance over separate baseline models.
- We discuss decision-making criteria and highlight the practical significance in context of wind turbine fault detection.

**Fig. 1:**
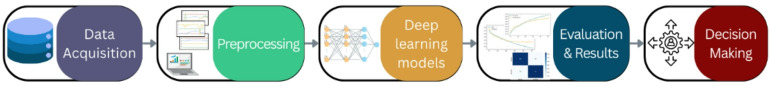
Summary of the proposed methodology.

The paper is structured as follows: Sect.  2 summarizes the related work, Sect.  3 goes through the methodology, Sect.  4 goes over the training configurations, Sect.  5 presents the results, and Sect.  6 discusses the implications and concludes the research. A summary of the research process is shown in Fig. 1.

## Literature review

The growing public demand and government efforts to lessen dependency on fossil fuels are causing a shift towards green energy. Wind power is the largest source of variable renewable electricity today and rapidly growing, making the reliability and efficiency of wind turbines essential. Fault detection in wind turbines is crucial to reduce costs, prevent failures, and ensure continuous operation. Blade failures can result in significant financial losses, extended downtime, and pose serious risks to the operational efficiency of wind farms^10^.

Rizk et al. investigated the integration of hyperspectral imaging and 3D convolutional neural networks (CNN) to detect defects such as cracks, erosion, and dirt accumulation^11^. This captures both spatial and spectral features. Roelofs et al. Explored transfer learning techniques within autoencoders to improve fault detection in situations where labeled data is scarce^12^. This method leverages pre-trained models to accelerate learning and generalization across wind turbine types. While data-driven models demonstrated better performance than the traditional physics-based models, they lacked interpretability, which raised concerns about their reliability. Letzgus et al. proposed an explainable AI framework for modeling wind turbines power curve with the aim to enhance the transparency and robustness of wind turbine machine learning models^13^. It uses novel metrics to measure alignment with the underlying physical principles and utilizes explainable AI methods for insights into turbine operation.

According to research, yaw system’s–the system in charge of orienting the wind turbine’s rotor to keep the blades facing the wind-failure rate can be as high as 6.7%, accounting for 13.3% of wind turbine downtime overall. In their work, Song et al. focused on addressing the challenges in fault detection in wind turbines yaw systems^14^. They introduced a novel technique that integrates LSTM neural network with self-attention mechanism. The proposed model was trained using data extracted from SCADA units of wind turbines. Asy’Ari et al. proposed a hybrid framework that combines bispectrum image analysis with CNN, CNN-LSTM, and CNN-BiLSTM designs for wind turbine gearbox fault detection^15^. Using this method the authors were able to effectively identify complex non-linear features. Another hybrid model was presented by Ma et al.^16^. The hybrid model integrates three components: 1D CNN, BiLSTM, and AdaBoost ensemble algorithm to enhance fault detection of wind turbine blades. The model was trained on multi source data and was able to capture both local patterns and long-term dependencies in the signals. Wang et al. introduced a multisensor aware deep learning framework for fault diagnosis for incipient inter-turn short circuits (ITSC)^17^. The method implements several deep learning architectures to detect seven types of ITSC faults by utilizing a combination of data from multiple sensors. Lee et al. proposed a framework that combines multiple machine learning and optimization techniques^18^. The framework addresses data imbalance using synthetic minority over-sampling technique (SMOTE) and under sampling techniques and incorporates physics-informed deep convolutional neural network (PDCNN) that uses domain knowledge to extract important features. Extracted features are then fed into XGBoost model optimized using an adaptive elite particle swarm optimization (AEPSO). Dai et al. presented a novel approach to fault diagnosis in wind turbines that combine kNN, enhanced with Deep Metric Learning (DML) and GANs^19^. In this approach data is preprocessed, then, GAN extracts generalized features. Finally, the optimized features are classified using kNN, achieving accurate real-time fault detection. Guo et al. developed a hierarchical framework for wind turbine damage detection that has two main steps^20^. The first step is the Haar-AdaBoost step for detecting regions of proposed damaged areas using haar-like feature extraction and AdaBoost classifier. The second step is a CNN classifier for classification of the detected region as either healthy or damaged and identification of the type of damage, if any.

Zhu et al. produced a method that combines long short-term memory (LSTM), fuzzy synthesis (FS), and transfer learning to predict the operational state of wind turbine gearbox (WTG) using SCADA data^21^. The study uses multidimensional LSTM-FS method, and variety of transfer learning algorithms. The produced operational state calibration framework can effectively detect the potential fault information of the WTG. Jia et al. utilized optical-thermal video data to inspect wind turbines damage while they’re operating^22^. The authors proposed AQUADA-Seg model. The model employes encoder decoder architecture that merges thermal and optical data. The integration of both types of data enhances segmentation. The model also includes a memory component intended to leverage temporal information from video frames. C Davis et al. compared YOLO & Masked R-CNN two of the most prominent object detection architectures to determine which algorithm is best suited to detect some of the common wind turbine blade defects^23^. The authors also explored modifying Masked R-CNN using ResNet18-FPN to reduce computational complexity. Zhang et al. applied an AI-based operations (AIOps) hybrid approach called LSTM-AVAGMM that combines variational autoencoder, LSTM layers, and Gaussian mixture model^24^. The study utilizes SCADA data for anomaly detection, root cause analysis, and incremental training.

Sun et al. developed a spatial-temporal multi-learner neural network approach to perform fault detection with imbalanced wind turbines SCADA data^25^. Their proposed model integrates convolutional (CNN), recurrent (BIGRUs) layers, and adaptive learner selection. Liu et al. proposed a condition monitoring network called the Spatio-Temporal Graph Neural Network (STGNN) that utilizes graph convolution networks and gated recurrent units to model spatial and temporal dependencies of state variables and extract high-level features^26^. Ran et al. described an improved attention and feature balanced YOLO (AFB-YOLO) algorithm that builds upon the YOLO framework and incorporates weighted feature fusion, coordinate attention module to improve object representation, and redesigned efficient intersection over union (EIoU) function for better localization accuracy^27^. Bielecki et al. presented a fully unsupervised real-time wind turbine monitoring system that integrates adaptive resonance theory (ART-2) neural network with Gaussians mixture models to analyze data, detect faults and adapt without the need for prior training data^28^. Sheiati et al. explored turning this classification problem into similarity learning problem^29^. They presented a Siamese CNN-based method to identify blades across drone images taken at different times and combine it with deep learning-based segmentation to support blade tracking during operation. Lin et al. developed a deep learning-based early fault prediction system for wind turbines^30^. They utilized five years of operational data from 31 wind turbines in Taiwan, including variables such as temperature, wind speed, power generation, and rotor speed. Their methodology consisted of first applying random forest to select key features, then using an LSTM neural network to analyze time series data and predict failures.

Silva et al. analyzed the audible noise emitted from turbine components to detect faults in wind turbines^31^. Their approach uses unsupervised learning and image processing to detect abnormal noise using spectrogram analysis. Which in turn triggers a supervised classifier to alert operators of potential malfunctions. Manshadi et al. investigated the feasibility and performance of an offshore hybrid renewable energy system that consists of vortex bladeless wind turbines (VBTs) and Searaser wave energy converters (WECs) with the aim of predicting the net power using machine learning and deep learning techniques^32^. Jamil et al. proposed a novel deep boosted transfer learning technique for wind turbine gearbox malfunction identification, addressing the problem of negative transfer when utilizing data from various machines and working conditions^33^. By dynamically adjusting the weights of source and target samples during training, their instance-based method reduces the impact of irrelevant source data and concentrates learning on the most pertinent information. Ahmed et al. introduced a smart anomaly detection system for industrial machines utilizing a deep autoencoder framework to analyze vibration signals from gearboxes^34^. Trained on wind-turbine gearbox dataset, automatically extracts features from time and frequency domains without manual feature engineering, enabling early detection of faults such as broken gear teeth.

Multiple recent studies have explored the effectiveness of hybrid CNN-LSTM architectures in fault detection and anomaly monitoring in wind turbines. For instance, studies such as Kang et al.^35^, where the authors proposed a hybrid CNN-LSTM architecture optimized with the Adam algorithm to diagnose faults in the gearbox of point absorber wave energy converters; Zhang et al.^36^ who introduced a cascade CNN-LSTM architecture deep learning model for early abnormality detection in wind turbine main bearings. The model predicts the expected bearing temperature, deviations between the expected and actual temperatures signals faults; and Qi et al.^37^ who used a similar hybrid approach to detect and diagnose bearing faults in wind turbines using vibration signal data, have demonstrated the promise of such models in this domain. Based on these foundational studies, our study offers its own contribution, constructing a hybrid CNN-LSTM architecture enhanced with an attention fusion mechanism specifically for multivariate SCADA datasets from multiple wind farms. The model implements application-specific architectural modifications, finetuning, and training procedures that are suited for the specific fault types and nature of the data studied.

Advances in Transformer-based models and meta-learning approaches have shown strong potential in time-series forecasting and fault detection. Maldonado-Correa et al. applied two deep learning architecture based on transformers: Anomaly Transformer (AT) and TranAD, which learn normal behavior and detect deviations signaling faults^38^. Raju et al. proposed a novel machine learning model named HARO (Huber Adam Regression Optimizer)^39^. This model utilizes transformers network, Lasso Regression, and the Adam optimizer to capture complex sensor data and predict faults early to minimize downtime and optimize maintenance. Dwivedi et al. introduced an attention-based deep learning framework that utilizes a vision transformers model (ViT) to inspect and classify surface defects in renewable energy assets (wind turbines & solar PV panels) using drone captured high-resolution images^40^. Qiao et al. proposed a novel fault detection framework for wind turbine generators by combining a 1DCNN with Model-Agnostic Meta-Learning (MAML)^41^. Their approach enables early fault identification by converting SCADA into multiple tasks for meta-training, optimizing with both first and second-order MAML gradients, and selecting relevant features via Maximum Relevance Minimum Redundancy (mRMR). Wang et al. introduced a novel architecture called Online Soft-Label Meta-Learning with Gaussian Prototype Networks (SL-GPN) that combines meta-learning’s rapid adaptation with Gaussian prototypes for uncertainty modeling and dynamic soft labels to mitigate label errors^42^. Table 1 highlights and summarizes the strength and challenges of each of these algorithms.

**Table 1:** Summary of algorithms, strengths, and challenges in wind turbines fault detection.

Authors	Algorithm	Strength	Challenges
Rizk et al. (2024)^11^	Hyperspectral imaging + 3D Convolutional Neural Networks (3D CNN)	Captures spatial and spectral features; high accuracy	High-dimensional data processing; requires hyperspectral imaging equipment
Roelofs et al. (2024)^12^	Transfer learning with autoencoders	leverages pre-trained models for generalization across turbine types	Dependence on quality of pre-trained models
Letzgus et al. (2024)^13^	Explainable AI framework for power curve modeling	Enhance transparency and robustness	Complexity in developing explainable models
Song et al. (2025)^14^	Self-Attention Mechanism and LSTM	High accuracy and generalization ability	Depends on the quality, volume, and labeling of SCADA data
Asy’Ari et al. (2025)^15^	Bispectrum image analysis and CNN, CNN-LSTM, CNN-BiLSTM	Effectively capturing the gearbox failures.	Hybrid models are complex to implement and tune.
Ma et al. (2025)^16^	Hybrid 1D CNN-BiLSTM-AdaBoost	Leverage data from multiple sources	Without careful tuning, overfitting may occur
Wang et al. (2025)^17^	FCNet-5	High diagnostic accuracy and enhances reliability by incorporating multiple sensor	Struggles to differentiate between fault types with minute variations.
Lee et al. (2025)^18^	PDCNN + AEPSO-XGBoost Framework	Incorporates physical knowledge and addresses the issue of class imbalance in SCADA datasets	Computationally intensive and could have a risk of overfitting with noisy data
Dai et al. (2024)^19^	DFD-kNN	Computational efficiency due to the reduced data redundancy	Parameter sensitivity and dependency on historical data
Guo et al. (2021)^20^	Hierarchical framework: Haar-AdaBoost for region detection + CNN for classification	Efficient detection and classification of damage types	Complexity in multi-step processing
Zhu et al. (2022)^21^	LSTM + Fuzzy Synthesis + Transfer Learning for gearbox operational state prediction	Effective fault detection using SCADA data	Requires extensive SCADA data
Jia et al. (2024)^22^	AQUADA-Seg	Enhanced segmentation by integrating thermal and optical data	requires synchronized multi-modal data
Davis et al. (2024)^23^	YOLO and Mask R-CNN	Real-time object detection	Trade-off between accuracy and speed
Zhang et al. (2024)^24^	LSTM-AVAGMM	Root cause analysis and incremental training	Complexity of hybrid model
Sun et al. (2023)^25^	Spatial-temporal multi-learner neural network	Handles imbalanced SCADA data	requires careful learner selection
Liu et al. (2024)^26^	STGNN	Models spatial and temporal dependencies	Graph construction complexity
Ran et al. (2022)^27^	AFB-YOLO	Better localization accuracy	Requires careful tuning
Bielecki et al. (2021)^28^	Unsupervised real-time monitoring with ART-2 neural network + Gaussian mixture models	No need for prior training data	Potential sensitivity to noise - method may have false alarms
Sheiati et al. (2024)^29^	Siamese CNN for blade identification + deep learning segmentation	Supports blade tracking over time	Segmentation accuracy affects tracking
Lin et al. (2024)^30^	Random forest for feature selection + LSTM for early fault prediction	Effective time series analysis	Needs extensive historical data
Silva et al. (2025)^31^	Image processing on audible noise spectrograms	Early fault detection via abnormal noise	Noise variability - requires good noise data quality
Manshadi et al. (2022)^32^	Offshore hybrid system power prediction	Predicts net power effectively	data availability
Jamil et al. (2022)^33^	Deep boosted transfer learning gearbox malfunction ID	Improves learning across conditions	Transfer learning challenges
Ahmed et al. (2023)^34^	Deep autoencoder on vibration signals for anomaly detection	Early fault detection without manual engineering	Needs vibration data
Kang et al.^35^	Adam-optimized CNN-LSTM	High diagnostic accuracy for gearbox faults through adaptive optimization.	Computational complexity and limited to controlled environments
Zhang et al.^36^	CNN-LSTM cascade model	Early fault detection using SCADA temperature data	Limited by low-frequency data.
Qi et al.^37^	CNN-LSTM vibration-based fault detection	High accuracy on vibration-signal-based bearing fault detection	Relies on clean, high-quality data and is computationally intensive.
Maldonado-Correa et al.^38^	Anomaly Transformer and TranAD,	Use self-attention mechanisms to capturing long-range dependencies and complex temporal patterns	Computational limits; only up to 18 variables could be processed due to resource constraints
Raju et al.^39^	HARO	High prediction accuracy and effective feature selection using Lasso regression	Computational complexity and dependency on high quality data
Dwivedi et al. (2024)^40^	Attention-based Vision Transformers (ViT) on drone images	attention mechanism improves focus on relevant features	Large data requirements and computationally intensive
Qiao et al.^41^	1DCNN with Model-Agnostic Meta-Learning	Meta-learning enables rapid adaptation	Experimental validation is limited to one wind farm
Wang et al.^42^	SL-GPN	Addresses real-world challenges like small-sample and label error problems	high computational complexity

## Methodology

Despite significant advancements in wind turbine condition monitoring, developing reliable fault detection systems remain challenging due to the high dimensionality of sensor data, class imbalance in anomaly events, and the scarcity of publicly available datasets that capture diverse failure modes^43,44^. Existing methods are challenged to generalize across heterogeneously diverse wind farm operating conditions, particularly when sensor configurations are different, operating scenarios are different (e.g., onshore/offshore), or anomalies are rare and scenario specific^7,45^. In this paper, these constraints are addressed by integrating multiple datasets from different wind farms in order to build a more robust and diverse dataset that will be able to generalize better and explore diverse deep learning techniques.

### Dataset description

Experiments in this paper are performed using a benchmark standard dataset that is publicly available^43^. It contains 95 high-dimensional time series data sets collected over a period of 89 years from 36 wind turbines across three wind farms A, B, and C. Wind farm A is an onshore wind farm in Portugal, for which the data are accessed from the EDP-open data platform, while wind farms B and C are offshore wind farms based in Germany. The data sets are anonymized to preserve the confidentiality of the locations and assets of the wind farms. Each data set applies to a single event for one specific wind turbine and exists in a CSV format. The data sets contain 86, 257, or 957 features for every wind farm, capturing 10-minute means of the sensor measurements, with sometimes also the minimum, maximum, and standard deviations of the sensors. Other columns hold anonymized timestamps, asset IDs, operational status, and event labels to distinguish anomaly events (prior failures) and usual events. The whole dataset is well-balanced with 44 records as anomaly and 51 as normal behavior. In-depth metadata files hold event information and feature descriptions for enabling in-depth analysis and modeling of wind turbine operating states and fault prediction. Table 2 shows a summary of the dataset.

**Table 2:** Dataset description.

	Turbines	Anomaly events	Normal events	Total events	Sensors	Features
Wind Farm A	5	11	11	22	54	54
Wind Farm B	9	6	9	15	63	257
Wind Farm C	22	27	31	58	238	957

### Data preprocessing

The preprocessing pipeline was designed to prepare the raw wind turbine sensor data for effective fault detection modeling. The procedure is described as follows: The data set contains three sub-datasets related to three separate wind farms; Farm A, Farm B, and Farm C, each with different sensor configurations and sets of features. The data sets were loaded individually, together with their feature description files, to support informed feature selection and harmonization.

#### Feature selection and enhancement

To ensure data consistency across the heterogeneous datasets, we compared the three datasets and selected the most relevant and common features of all the datasets. The selection process was based on two main criteria: first, domain knowledge and relevance to the wind turbine faults; and second, identification of common features and confirmation that their definitions, sensor types, units, and measurement scales were consistent and identical across all three farms to ensure comparability.

This process resulted in the selection of the following four key features critical for fault detection:

1. Ambient Temperature: The temperature of the air surrounding the equipment, measured in degrees Celsius (°C). Thermal stress impacts the component lifespan; abnormal temperatures often precede failures.
2. Grid Frequency: The frequency at which the electrical grid operates, measured in Hertz (Hz). Fluctuations may reveal generator synchronization issues and power quality problems.
3. Active Power: The amount of real power delivered by the system, measured in kilowatts (kW). Deviation in active power signal performance is a sign of degradation or fault in energy output.
4. Rotational Speed: The speed at which a rotating component (such as a turbine shaft) spins, typically measured in revolutions per minute (rpm). Critical for bearing health assessment; abnormal vibrations or speeds often correlate with faults.

Table 3 shows the selected features, the corresponding sensor reading in each of the datasets and the unit of measurement. Rotational speed from Farm C was converted from radians per second to revolutions per minute (rpm) for consistency. Unit conversion for rotational speed (rad/s → rpm) ensured cross-dataset comparability and consistency.

**Table 3:** Selected features.

Feature	Farm A	Farm B	Farm C	Unit
Ambient_temperature	Sensor_0 (°C)	Sensor_8 (°C)	Sensor_7 (°C)	°C
Grid_frequency	Sensor_26 (Hz)	Sensor_23 (Hz)	Sensor_47 (Hz)	Hz
Active_power	Power_30 (kW)	Power_62 (kW)	Power_6 (kW)	kW
Rotational_speed	Sensor_18 (rpm)	Sensor_19 (rpm)	Sensor_8 (rads/s; must be converted to rpm)	Rpm (revolutions per minute)

In order to capture temporal patterns and seasonality inherent in the data, a number of features were added to the dataset including hour, day, and month. The hour of the day can reveal daily cycles, while the day and month can reveal weekly and seasonal trends. Including these features will increase robustness especially in complex or non-linear situations. This enhances model performance beyond what timestamps alone can provide.

#### Dataset integration

We acknowledge the potential influence of seasonal and temporal variations when integrating datasets collected in different years. To reduce the effects of variation and ensure comparability of the datasets, all the standard meteorological variables were normalized using the z-score method within dataset. The normalization is to center and scale each variable in its seasonal environment, which helps reduce the impact of seasonal variations and long-term trends.

To further improve the model’s sensitivity to seasonality effects, we engineered additional time-related features including month, day, and hour as mentioned in the previous section. These features provide explicit temporal context that enables models to capture seasonal patterns more effectively, thereby enhancing the robustness of fault detection tasks.

To build the most comprehensive and unified dataset, the selected features were extracted from the preprocessed datasets of the three farms then combined into a single unified dataset. This process resulted in a powerful, consolidated dataset containing 5,239,204 individual timestamped records that preserve event independence across farms while providing consistent feature space and temporal resolution for model training and evaluation.

While all events were normalized and aligned to be defined over the same features, we preserved the independence of events by including an event ID column in addition to timestamps. By including the event ID as part of our temporal data, we could sort by both events and timestamp to ensure that each event would happen in the correct order within each event. We used these two features to perform segmentation, then they were removed and used in the training.

During segmentation, overlapping windows were formed strictly within individual events. There was no mixing across events. Records from separate events were not temporally aligned as each record represents a separate, independent, and non-overlapping time period.

#### Handling missing values and timestamps

The combined dataset contained 288 missing values exclusively in the ambient temperature feature within event ID 36. These missing values were imputed by filling with zeros to maintain data integrity. All other features had no missing values. Wind turbine data were expected to be sampled at fixed 10-minute intervals. To ensure temporal completeness: Missing timestamps within each event were identified and inserted, resulting in a dataset expansion of approximately 0.6%. Missing sensor values in the newly inserted timestamps were imputed using zeros within each event to preserve continuity. Dataset completeness was quantified per event as the ratio of original to expected timestamps, ensuring data quality for subsequent modeling.

#### Standardization

To reduce the influence of outliers and ensure comparability across features, all selected features were standardized using z-score normalization^46^:

1 $$\:Z=\frac{x-\mu\:}{\sigma\:}$$

where:

- $\:Z$ is Z-score (standardized value).
- $\:\mu\:$ is the mean of the population.
- $\:\sigma\:$ is the standard deviation of the population.
- $\:x$ is the value being standardized.

**Figure:**
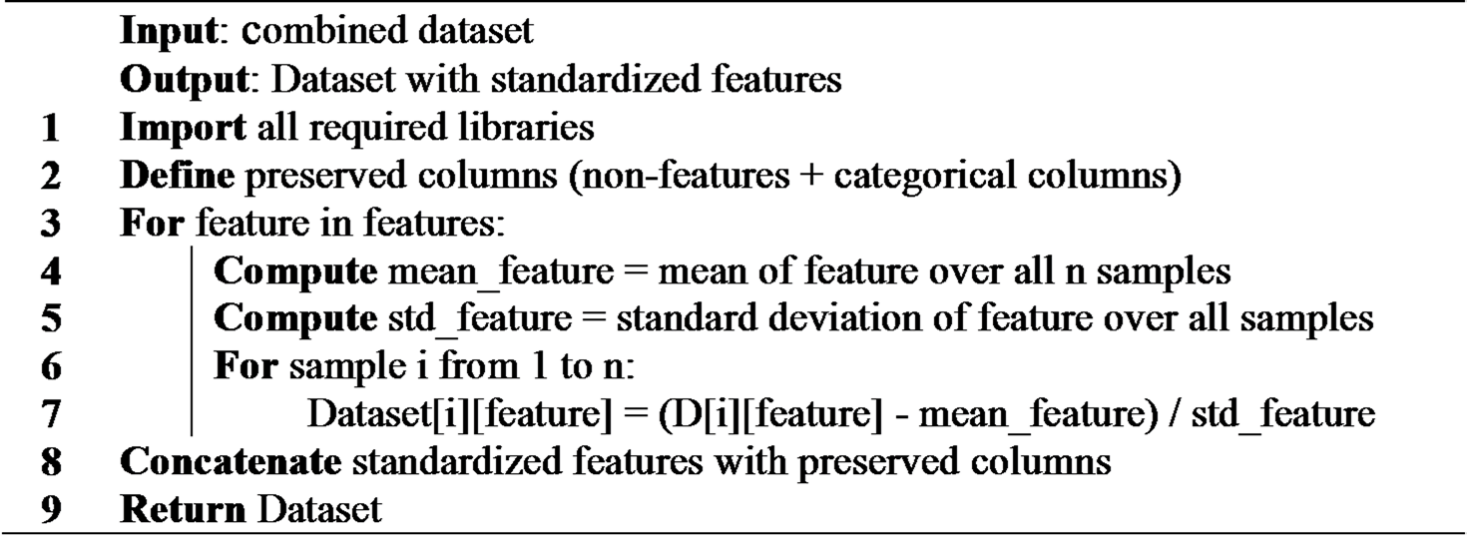
**Algorithm 1**: Standardization of the combined dataset.

To summarize, in order to achieve consistency among heterogeneous datasets, all meteorological variables were resampled to a common 10-minute interval, normalized through z-scores, and missing values were imputed as per guidelines provided in the dataset documentation. Similar preprocessing strategies have also been recommended in literature for the integration of multi-source energy data^47^.

#### Segmentation and labeling

The time series data were segmented into overlapping windows of 144 timestamps (equivalent to 24 h) with a 50% overlap (72 timestamps). Each segment was labeled as anomalous if more than 5% of its constituent timestamps were labeled as anomalies, otherwise as normal.

The datasets were not temporally aligned across wind farms and wind turbines since they originate from independent sources and cover different calendar periods. Rather than concatenating them arbitrarily after selecting the features, we treated each dataset as a separate input source during preprocessing, applied the same 10-minute resampling interval, concatenated the events into a single dataset, then normalized the features consistently. Then during segmentation, the data were segmented into windows carefully making sure every segment contains data from one event.

To mitigate class imbalance and increase the representation of anomaly samples, segmentation was refined by applying smaller overlap steps for anomalous data (36 timestamps, or 6 h) while maintaining a 72-timestamp overlap for normal data. This approach effectively doubled the number of anomaly segments.

**Figure:**
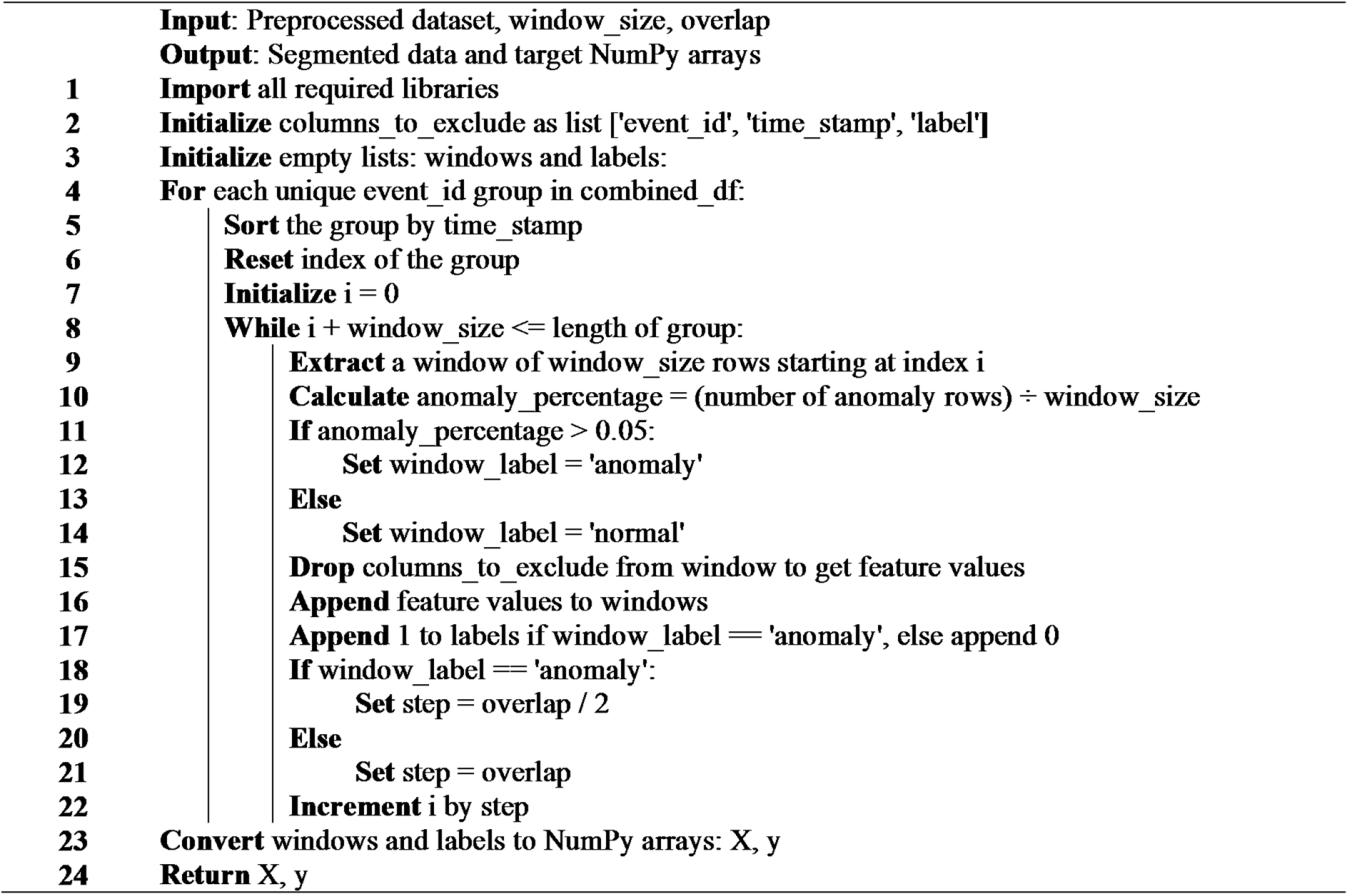
**Algorithm 2**: Segmentation of the dataset into time windows.

#### Data partitioning

**Fig. 2:**
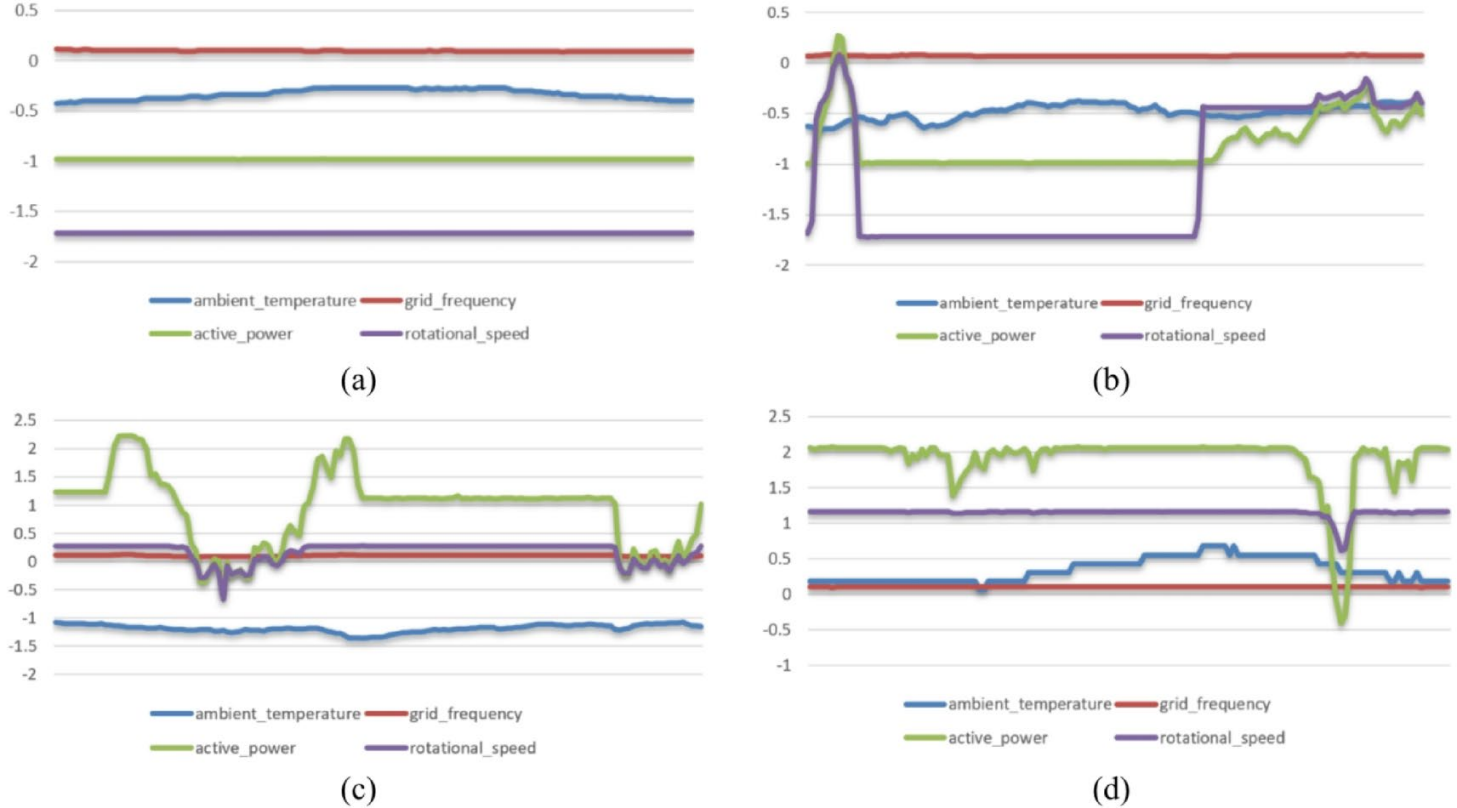
Data samples after preprocessing. (**a**,**b**) anomalous samples, (**c**,**d**) normal samples.

To train the models 6231 anomaly samples and 7000 normal samples were used. And weights were given to each class in order to fix the class imbalance. The data were split into three partitions: train set containing 70% of the data, validation set and test set each containing 15% of the data. Figure 2 shows visualization of the dataset samples after preprocessing.

### Deep learning models architecture

The main motivation of this experiment is to provide a uniform benchmark comparison to commonly used standard deep learning architectures all subjected to the same experimental conditions. We consider developing a fair and reproducible baseline a prerequisite task before considering the next step of dealing with greater complexities or otherwise uniquely modified architectures.

#### 1D CNN

**Model Architecture**

**Fig. 3:**
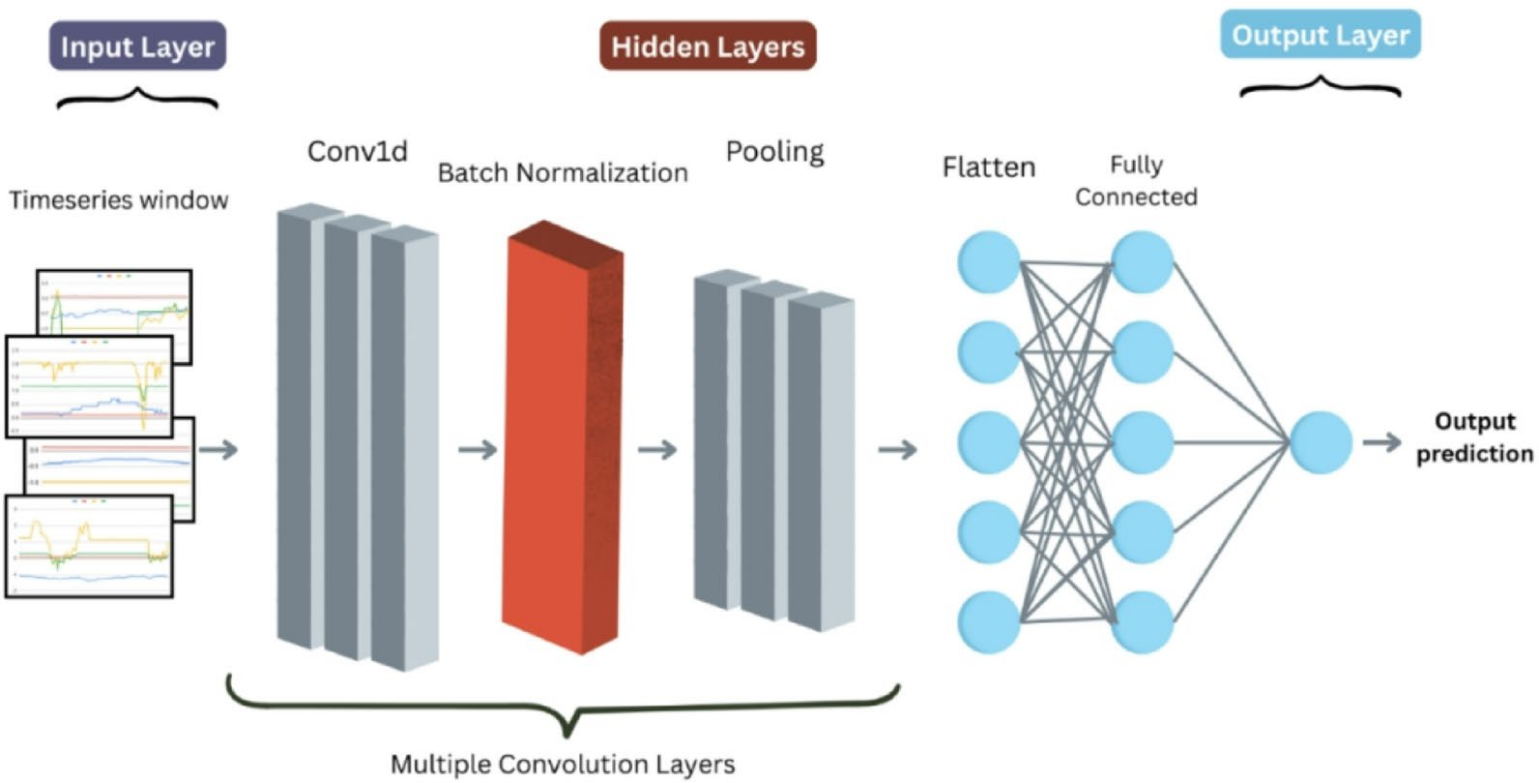
1D-CNN architecture.

We employed a one-dimensional convolutional neural network (1D-CNN) architecture tailored for timeseries classification (Fig. 3). The model consists of input layer of size 144 × 7 [m×n sequences of length n timesteps and m features]. The architecture includes two convolutional blocks, each comprising a 1D convolutional layer (with 48 filters and kernel sizes 9 each), followed by batch normalization, a tunable activation function (ReLU), max pooling (pool size 2). After flattening, the model includes one or two dense layers with L2 regularization and 0.2 dropout. The output layer is a single neuron with a sigmoid activation for binary classification. The model is trained using the Adam optimizer, and binary cross-entropy loss. Table 4 highlights the model hyperparameters.

**Table 4:** 1D-C3NN hyperparameters.

Hyperparameter	Value
Convolutional layers	3
Filters per layer	64/64/32
Kernel sizes	2
Activation function	ReLU
Pooling size	2
Convolutional dropout	0.1
Dense layers	2
Dense units	32
Dense dropout	0.3
L2 regularization	1e-05

This layered approach allows the model to learn hierarchical representations from sequential input data, leveraging the strengths of convolutional architectures in feature extraction and regularization.

**Figure:**
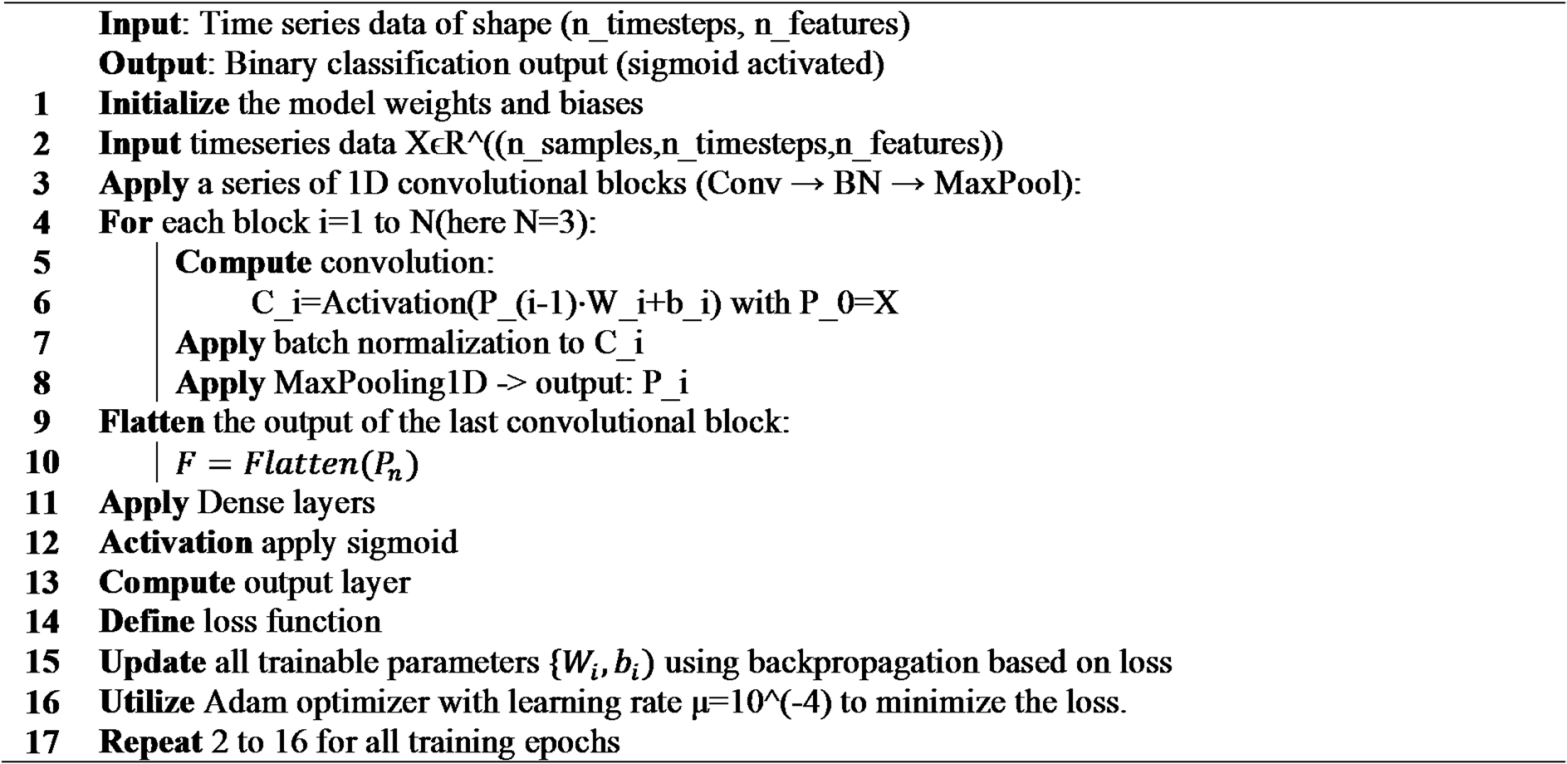
**Algorithm 3**: 1D-CNN.

#### TCN

**Model architecture**.

**Fig. 4:**
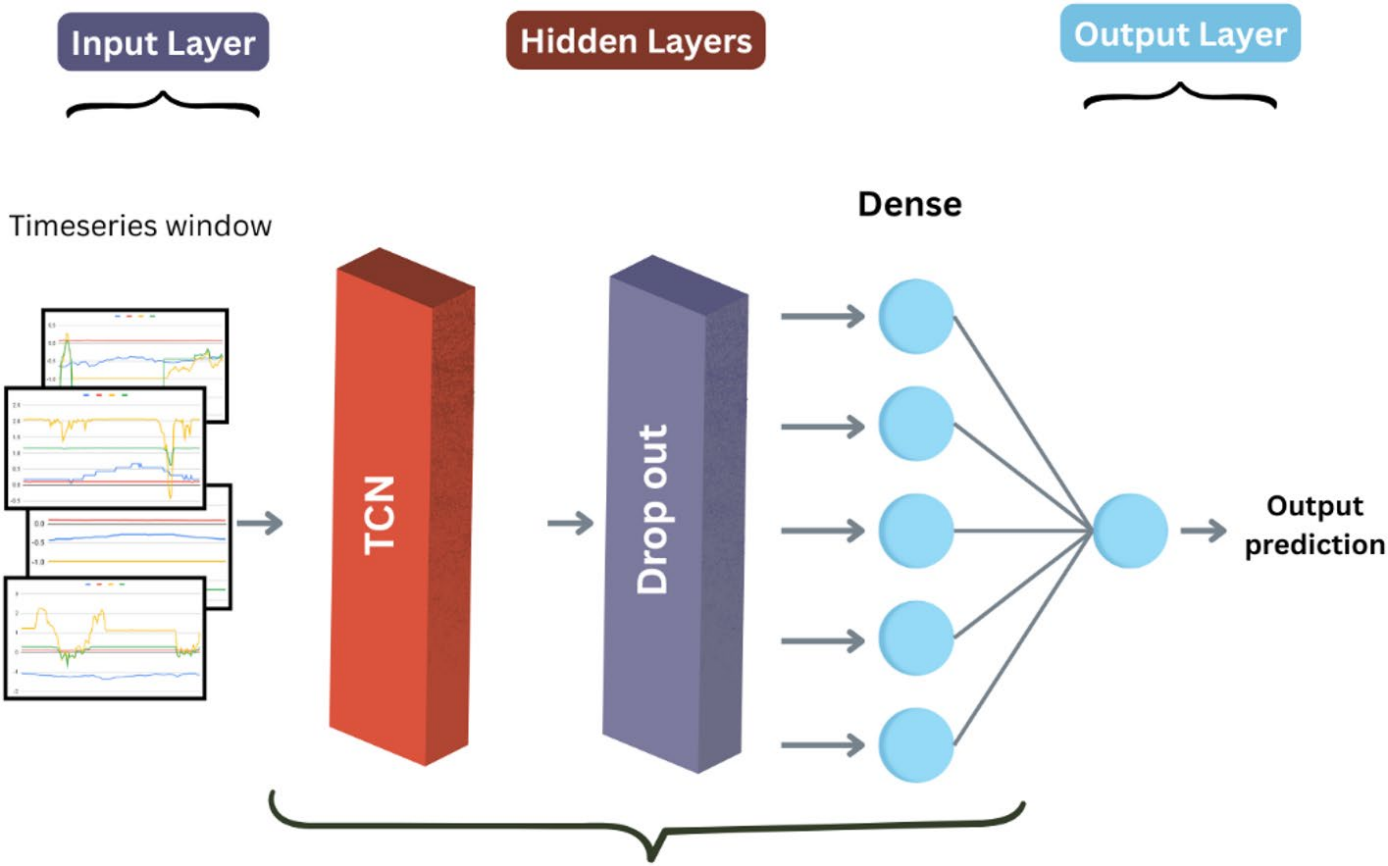
TCN architecture.

Temporal Convolutional Network (TCN) model is a recent deep neural network architecture specifically designed for sequence modeling^48^. It’s more accurate and computationally efficient than common traditional sequence models^49^. TCN capitalizes on two key components:

- Dilated Convolution: Applies the filters on the intervals, allowing the model to look further back in time with fewer layers.
- Residual Blocks: Help in training deeper networks by making it simpler for gradients to flow through.

which makes it suitable for time series classification tasks such as fault diagnosis from wind turbines. The used TCN model architecture (Fig. 4) employs 1D causal dilated convolutions to process input sequences with shape $\:({n}_{timesteps},\:{n}_{features})$, each containing 144 timesteps with 7 features at each step. The model begins with a TCN layer with 128 filters, 7 as the size of the kernel, and^1,2,4,8^ as the pattern of dilation for the model to capture long temporal patterns efficiently while preserving sequence order. This layer is followed by a dropout layer with rate 0.3 to avoid overfitting, a dense hidden layer of 32 ReLU-activated units, and an ultimate sigmoid-activated output neuron for binary classification. The model is then compiled using the Adam optimizer and binary cross-entropy loss function, optimizing for accuracy. This environment and hyperparameters shown in Table 5 allows TCN to learn complex temporal patterns in sensor readings, enabling stability and scalability for predictive maintenance applications.

**Table 5:** TCN hyperparameters.

Hyperparameter	Value
dilations	[1, 2, 4, 8]
Filters	128
Kernel sizes	7
Activation function	ReLU
Dropout	0.3
Dense layers	1
Dense units	32

**Figure:**
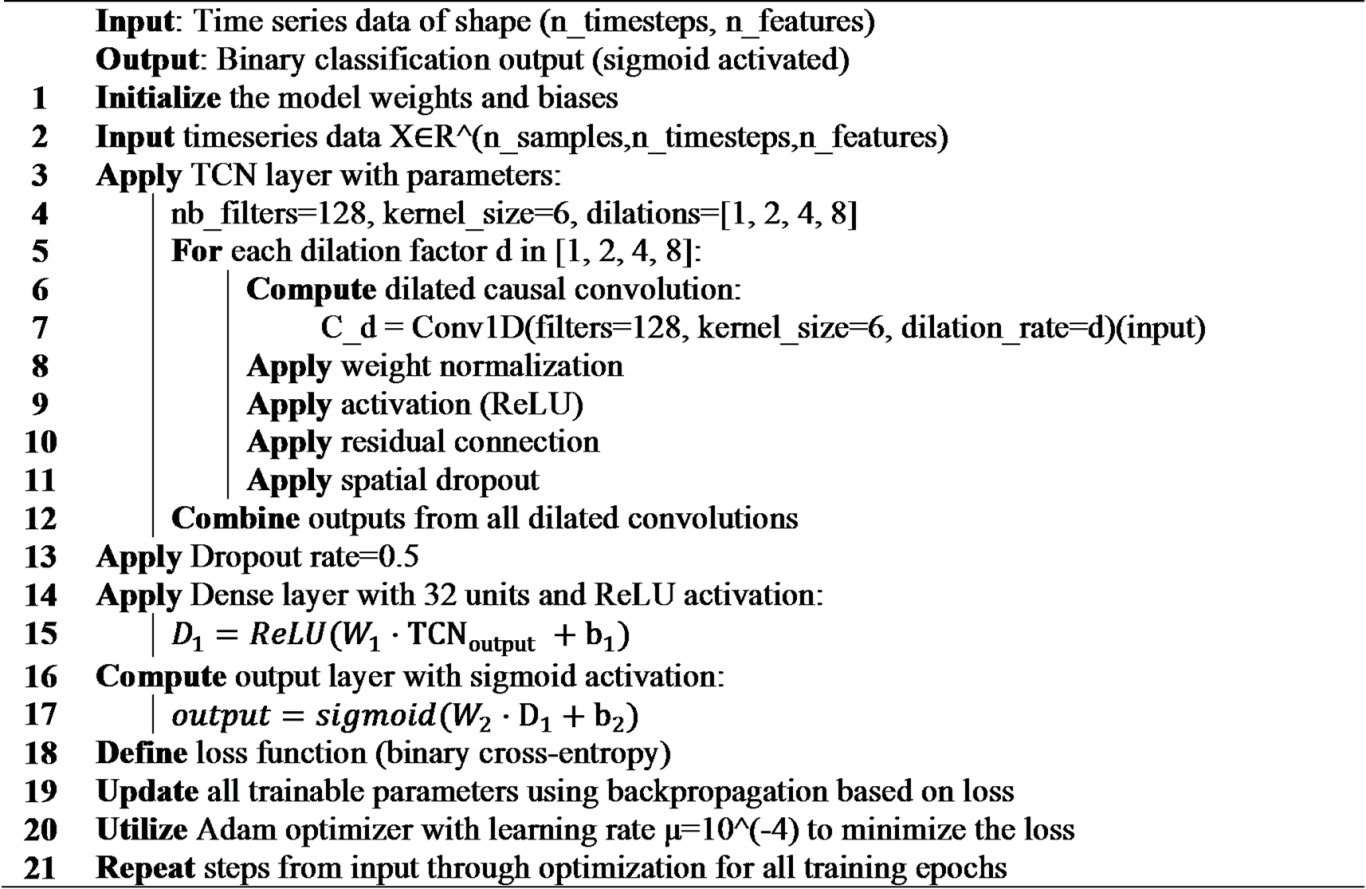
**Algorithm 4**: TCN.

#### RNN

**Fig. 5:**
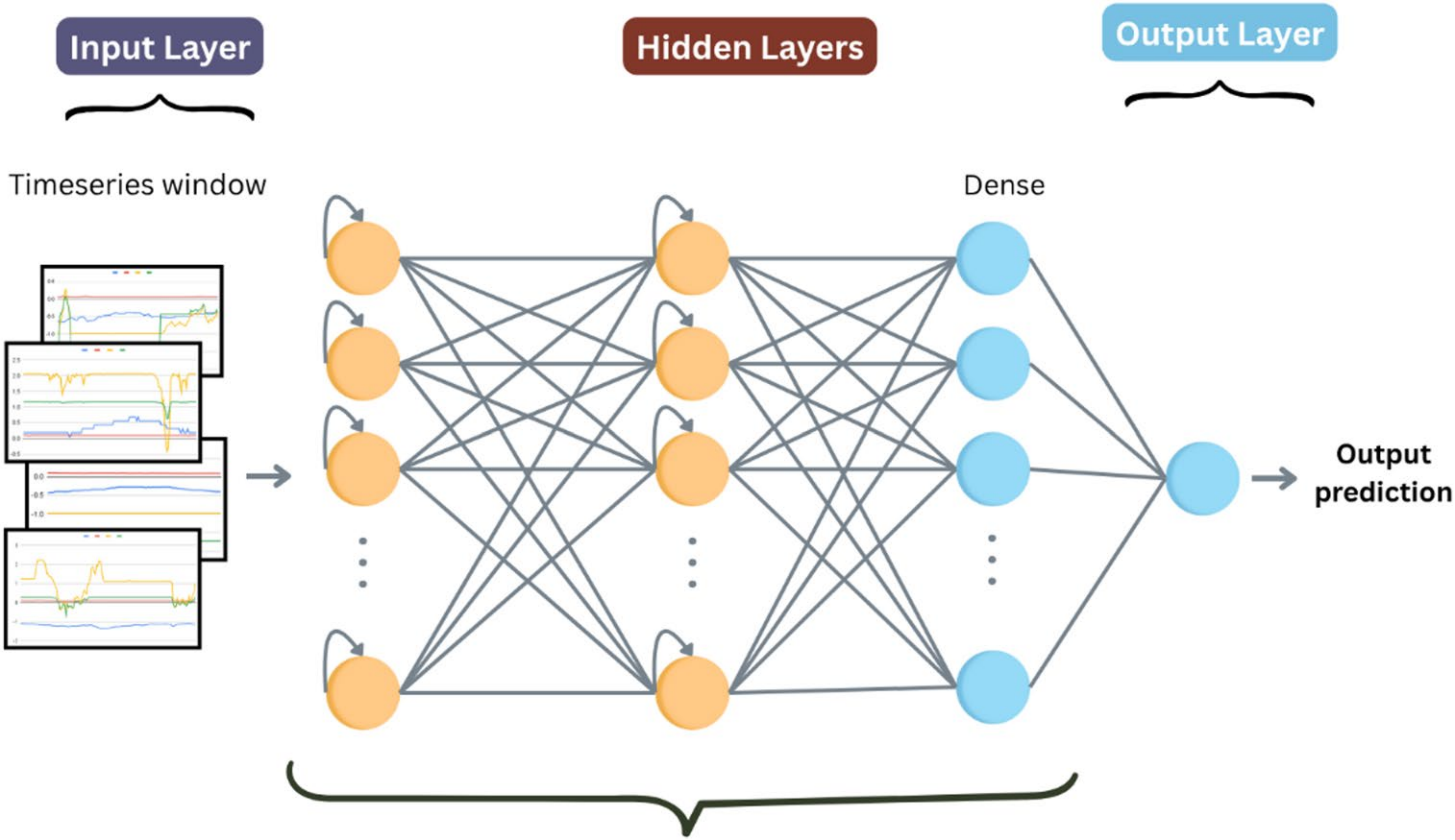
RNN architecture.

We developed a recurrent neural network (RNN) architecture optimized for time-series anomaly detection (Fig. 5). The model accepts input sequences of shape (timesteps × features) and processes them through the following layers:

1. Input Layer: Accepts sequences of 10-minute sensor measurements over a 24-hour window (144 timesteps).
2. RNN Blocks:

- Two stacked SimpleRNN layers with 160 and 128 units respectively, using tanh activation.
- Layer 1 returns sequences to feed into Layer 2; Layer 2 returns only the final output vector.
- L2 regularization applied to recurrent kernels.
- Dropout rate 0.1 between RNN layers to prevent co-adaptation.

3. Dense Block:

- A fully connected layer with 128 units and ReLU activation.
- L2 regularization (λ = 0.00077) and 30% dropout to enhance generalization.

4. Output Layer: A single neuron with sigmoid activation for binary classification (normal vs. anomaly).

The model was compiled using the Adam optimizer (learning rate = **0.001**) and binary cross-entropy loss, enabling efficient gradient-based learning of temporal patterns in turbine sensor data. Three strategies were used to ensure generalization:

1. Stratified Data Splitting: 15% of training data reserved for validation, 15% held out for testing.
2. Regularization:

- Layer-wise L2 regularization to constrain weight magnitudes.
- Dropout between RNN and dense layers.

3. Early Stopping: Training halted if validation loss did not improve for 5 consecutive epochs, preventing overfitting to training noise.

Hyperparameter tuning was performed using Keras Tuner Hyperband algorithms with the following key optimizations (Table 6):

**Table 6:** RNN hyperparameters.

Hyperparameter	Value
RNN layers	2
First layer filters	160
Second layer filters	128
Activation function	Tanh
Dropout rate	0.1
Dense units	128
L2 regularization	1e-05
Learning rate	1e-3

**Figure:**
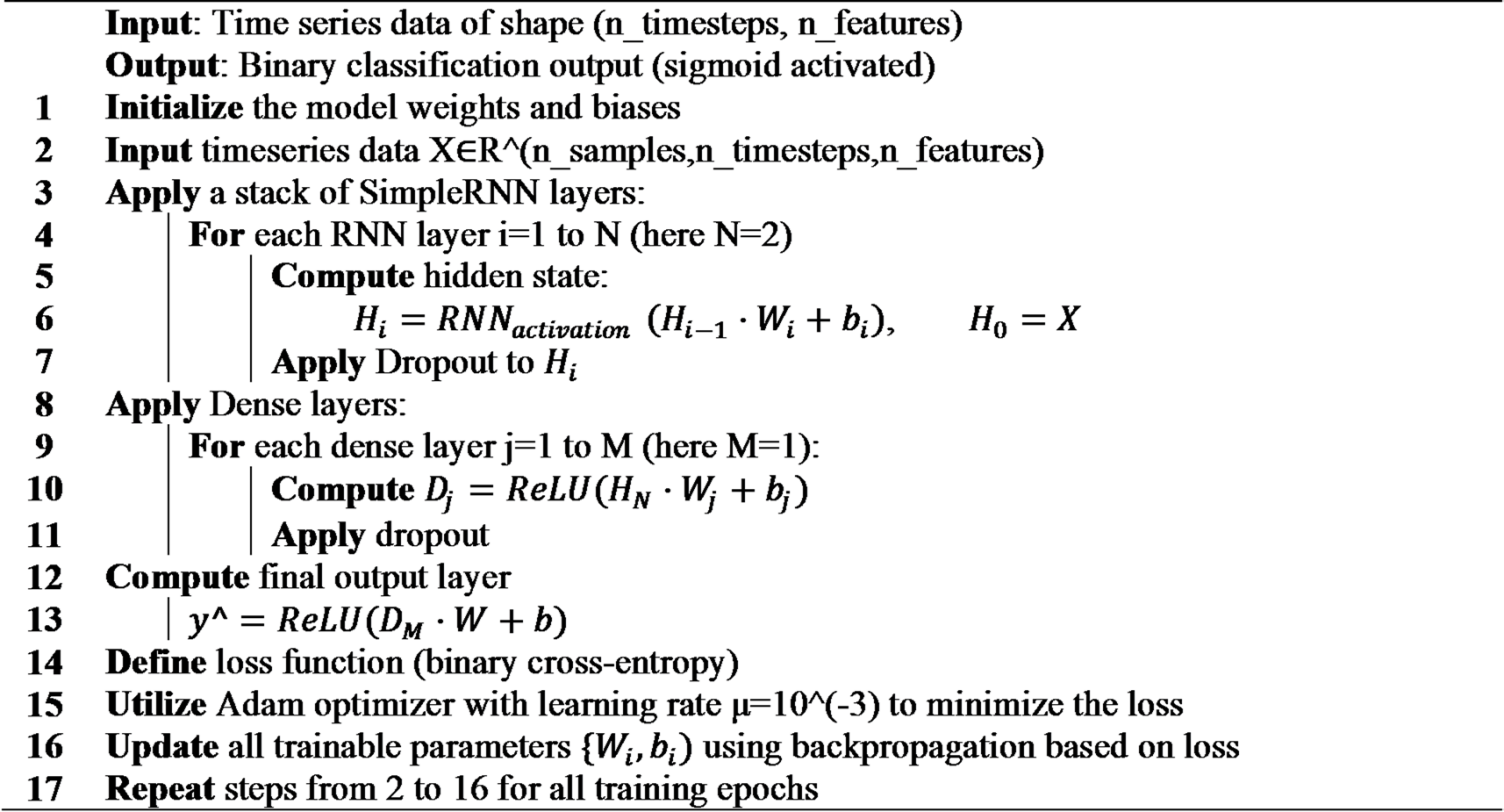
**Algorithm 5**: RNN.

#### LSTM

**Fig. 6:**
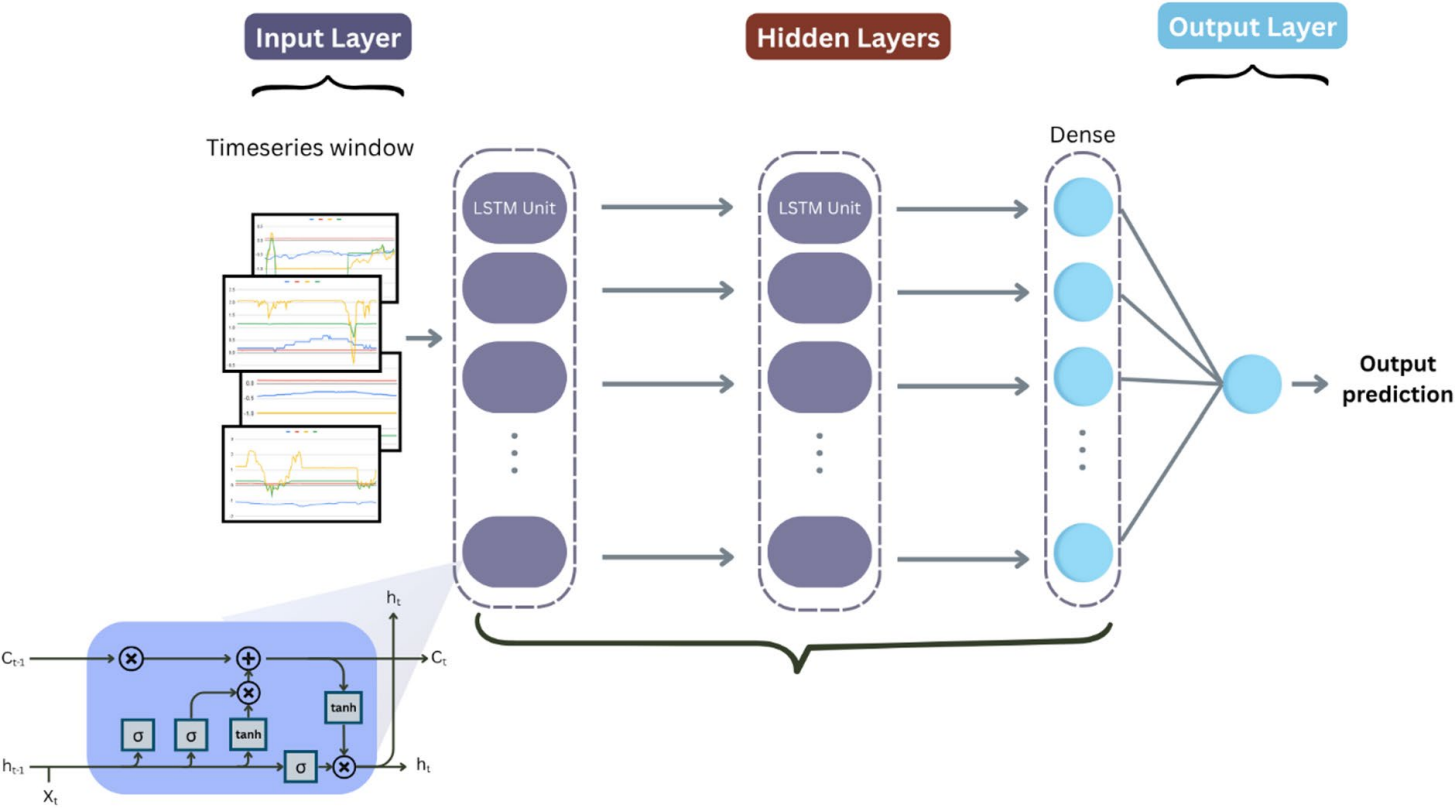
LSTM architecture.

We designed a stacked long short-term memory (LSTM) network to capture long-range temporal dependencies in wind turbine sensor data (Fig. 6). The architecture processes 24-hour sequences (timesteps × features) through the following components:

1. Input Layer: Accepts multivariate time-series windows of shape (n_timesteps× n_features).
2. LSTM Blocks:

- Three sequential LSTM layers with 128 and 128, and units respectively, using tanh activation.
- Intermediate layers return full sequences to enable deep temporal processing.
- Dropout applied after the LSTM layers.

3. Dense Block:

- A fully connected layer with 48 units and ReLU activation.

4. Output Layer: Single sigmoid-activated neuron for anomaly probability estimation.

The model was compiled using the Adam optimizer (learning rate = 0.001) with binary cross-entropy loss, optimized for sequential pattern recognition in SCADA data. Hyperparameters details are shown in Table 7.

**Table 7:** LSTM Hyperparameters.

Hyperparameter	Value
LSTM layers	2
First layer units	128
Second layer units	128
Activation function	Tanh
Dropout rate	0.1
Dense units	32
Dense activation	ReLU
L2 regularization	1e-05
Learning rate	1e-3

**Figure:**
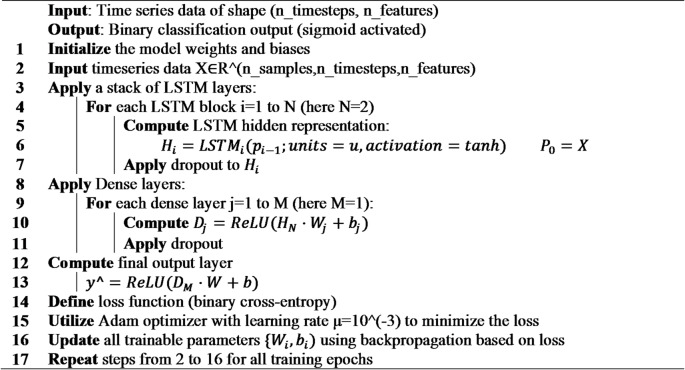
**Algorithm 6**: LSTM.

#### Bi-LSTM

**Fig. 7:**
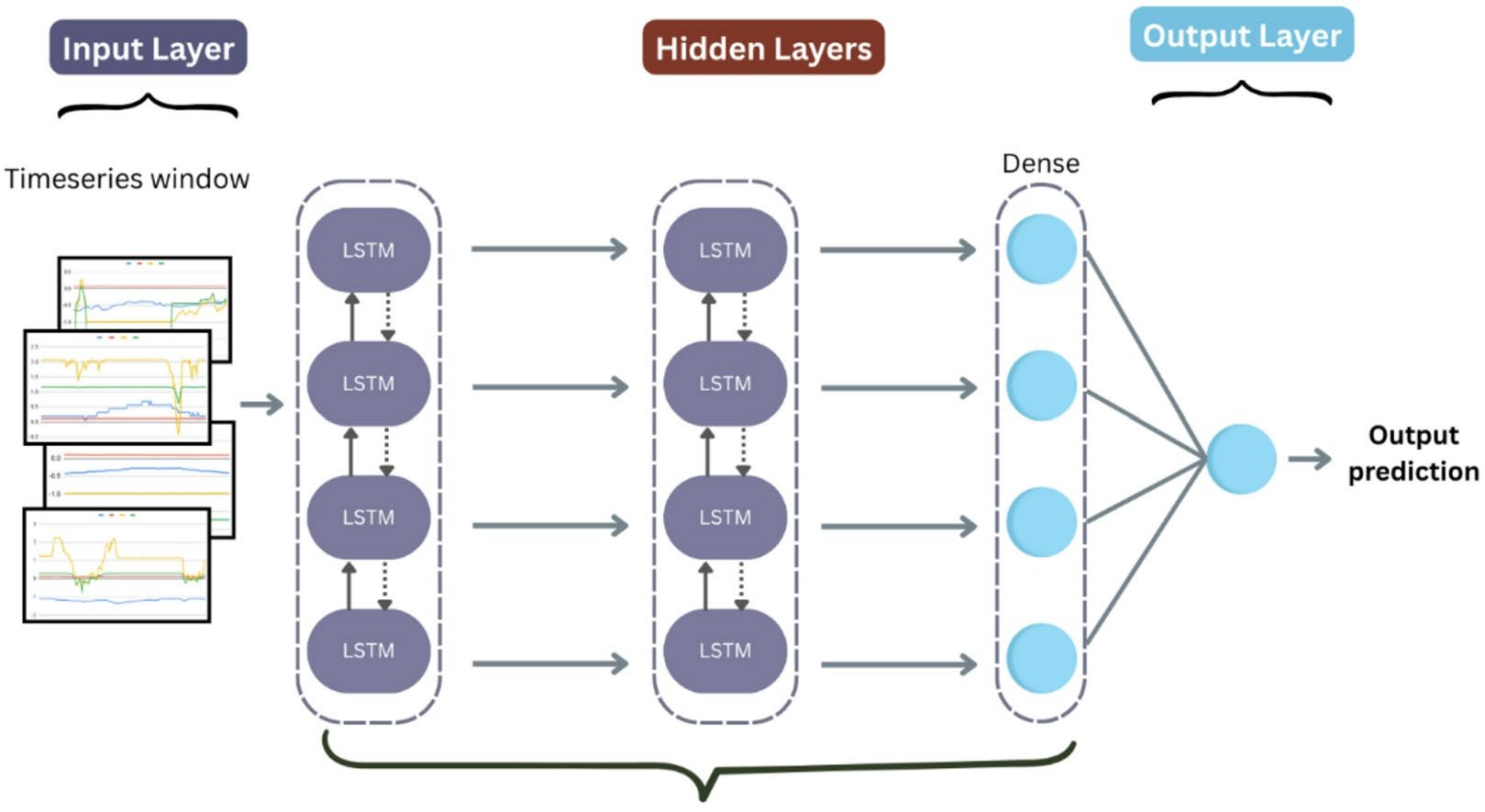
Bi-LSTM architecture.

We implemented a bidirectional long short-term memory (Bi-LSTM) network to effectively capture both past and future temporal dependencies in wind turbine sensor data sequences (Fig. 7). The model processes input sequences of shape (timesteps by features) through the following layers:

1. Input Layer: Accepts multivariate time-series windows representing 24 h of sensor readings.
2. Bi-LSTM Layers:

- Two stacked Bi-LSTM layers with 128 and 64 units respectively, each followed by dropout layers with rates of 10% to reduce overfitting.
- The first Bi-LSTM layer returns full sequences to feed into the second; the second returns only the final output vector.

3. Dense Layer:

- A fully connected layer with 32 units and ReLU activation to learn nonlinear combinations of extracted temporal features.

4. Output Layer: A single sigmoid neuron producing the anomaly probability for binary classification.

The model’s and hyperparameters, as detailed in Table 8, were carefully selected to ensure robust performance. The model was compiled using the Adam optimizer with a fixed learning rate of 0.001 and binary cross-entropy loss, optimizing the network for reliable anomaly detection in SCADA time series.

**Table 8:** Bi-LSTM hyperparameters.

Hyperparameter	Value
Bi-LSTM layers	2
First layer units	128
Second layer units	64
Activation function	Tanh
Dropout rate	0.1
Dense units	32
Dense activation	ReLU
L2 regularization	1e-05
Learning rate	1e-3

**Figure:**
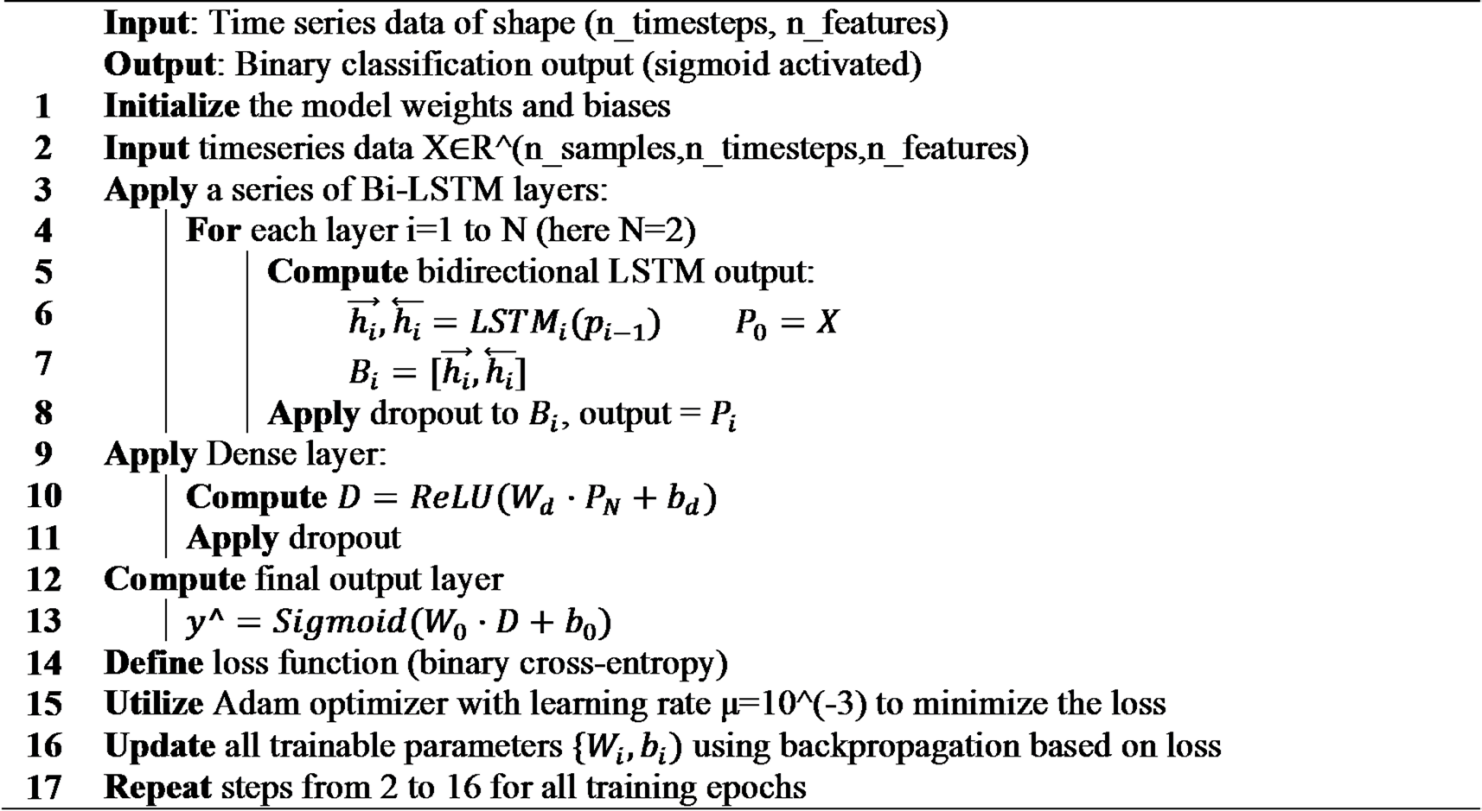
**Algorithm 7**: Bi-LSTM.

#### GRU

**Fig. 8:**
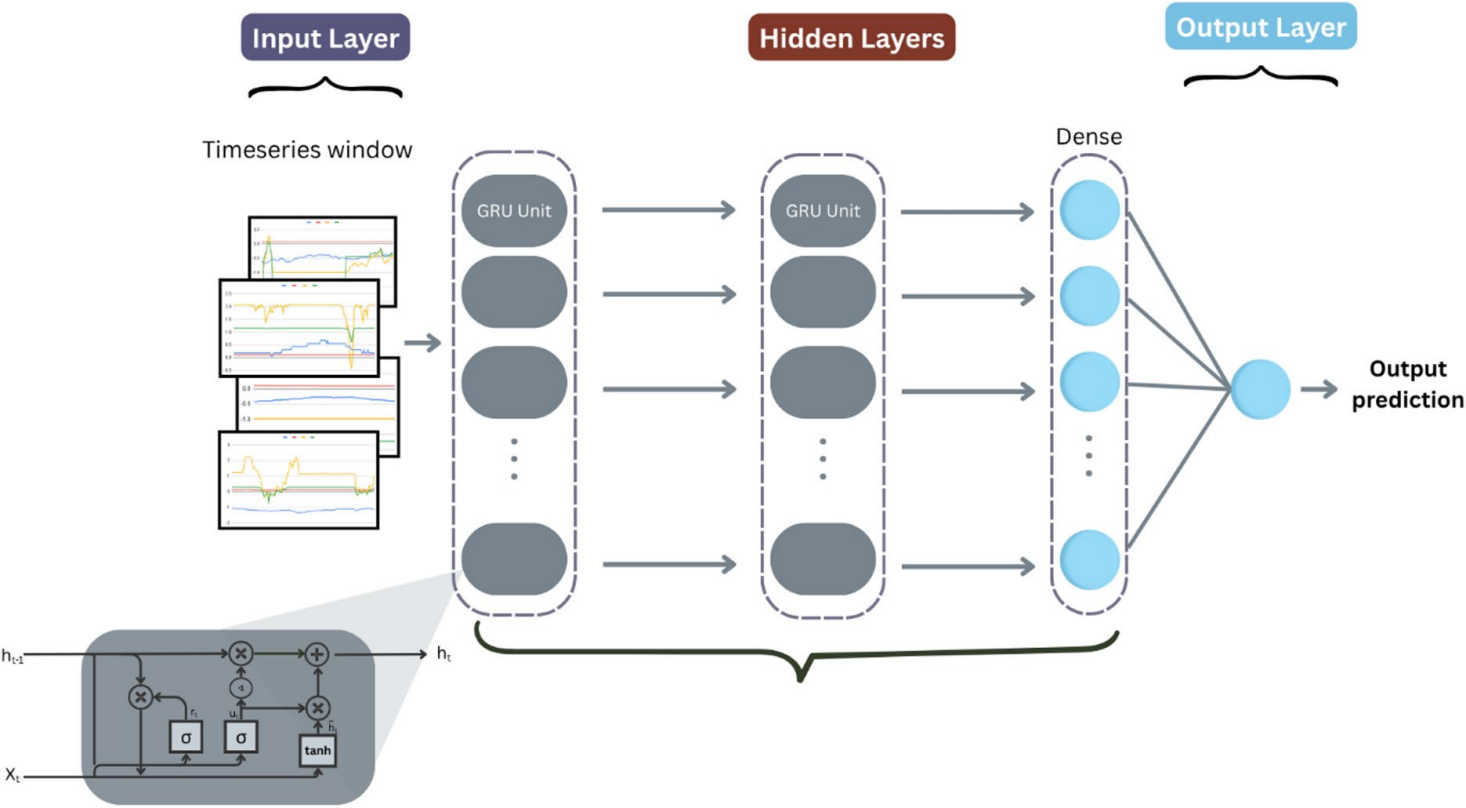
GRU architecture.

We implemented a gated recurrent unit (GRU) network tailored for early fault detection in wind turbine sensor data (Figure 8). The model processes input sequences through the following layers:

1. Input Layer: Accepts multivariate time-series windows representing 24 h of sensor measurements.
2. GRU Layers:

- Two stacked GRU layers with 128, and 64 units respectively.
- The first GRU layer return full sequences to enable deep temporal feature extraction; the final GRU layer outputs the last hidden state.
- Dropout with a rate of 30% is applied after each GRU layer to reduce overfitting.

3. Dense Layer: A fully connected layer with 32 units and ReLU activation to learn nonlinear combinations of temporal features.
4. Output Layer: A single sigmoid neuron producing the probability of anomaly for binary classification.

The model was compiled using the Adam optimizer with a learning rate of 0.001 and binary cross-entropy loss, optimizing for accurate and timely detection of wind turbine faults from SCADA data. Table 9 provides a detailed view of the model hyperparameters.

**Table 9:** GRU hyperparameters.

Hyperparameter	Value
GRU layers	2
First layer units	128
Second layer units	64
Activation function	Tanh
Dropout rate	0.3
Dense units	32
Dense activation	ReLU
L2 regularization	1e-05
Learning rate	1e-3

**Figure:**
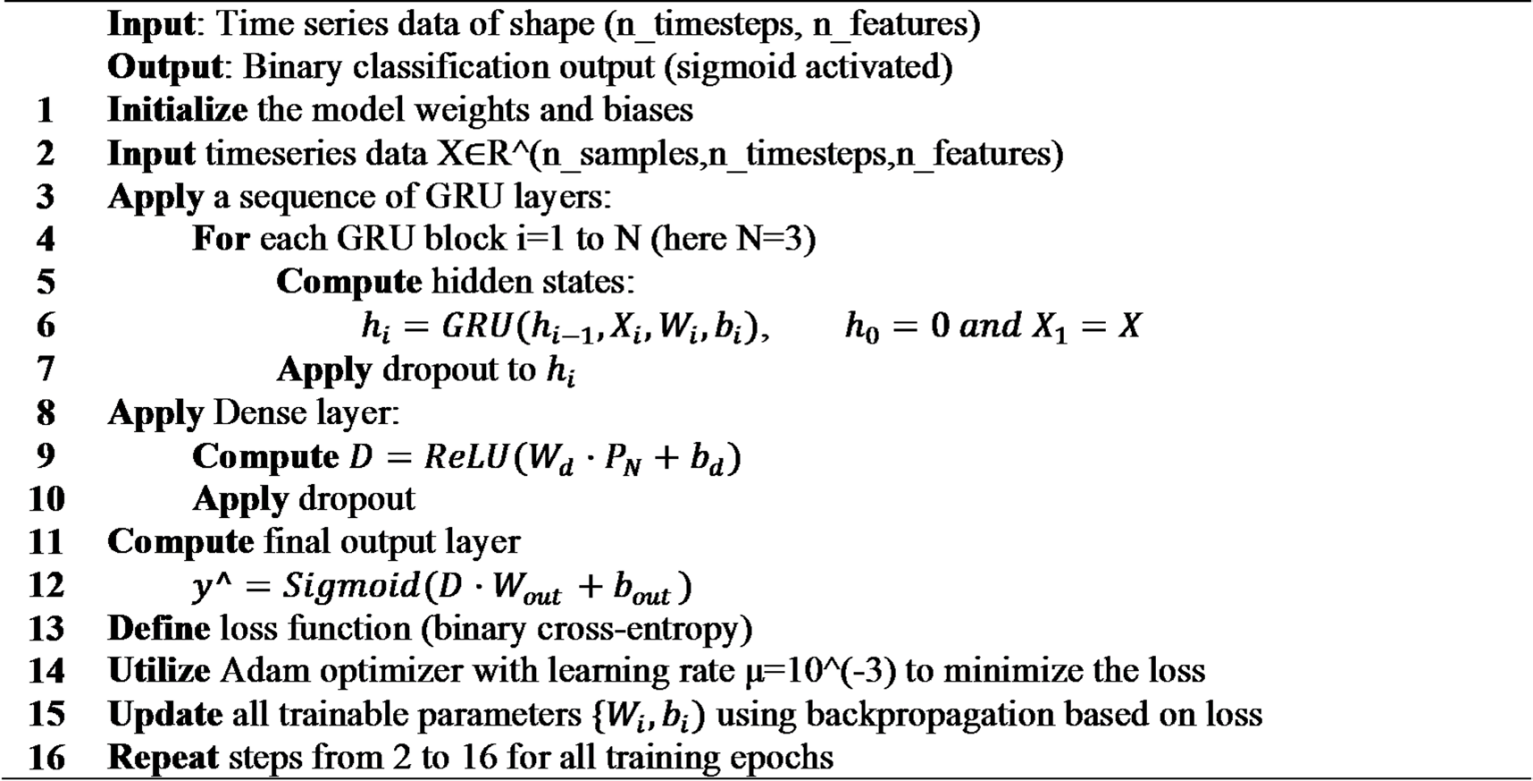
**Algorithm 8**: GRU.

### Hybrid CNN-LSTM with attention fusion

CNNs excel at extracting local, hierarchical features but they struggle when it comes to capturing long term dependencies in sequential data. On the other hand, RNNs, especially LSTMs, are well-suited at modeling temporal dynamics, but they might not be as suitable for capturing local, translation-invariant features. The fusion of these architectures has shown promise in addressing these limitations. Additionally, the integration of an attention mechanism allows the network to dynamically focus on the most relevant parts of the input sequence enhancing the model interpretability and performance.

**Fig. 9:**
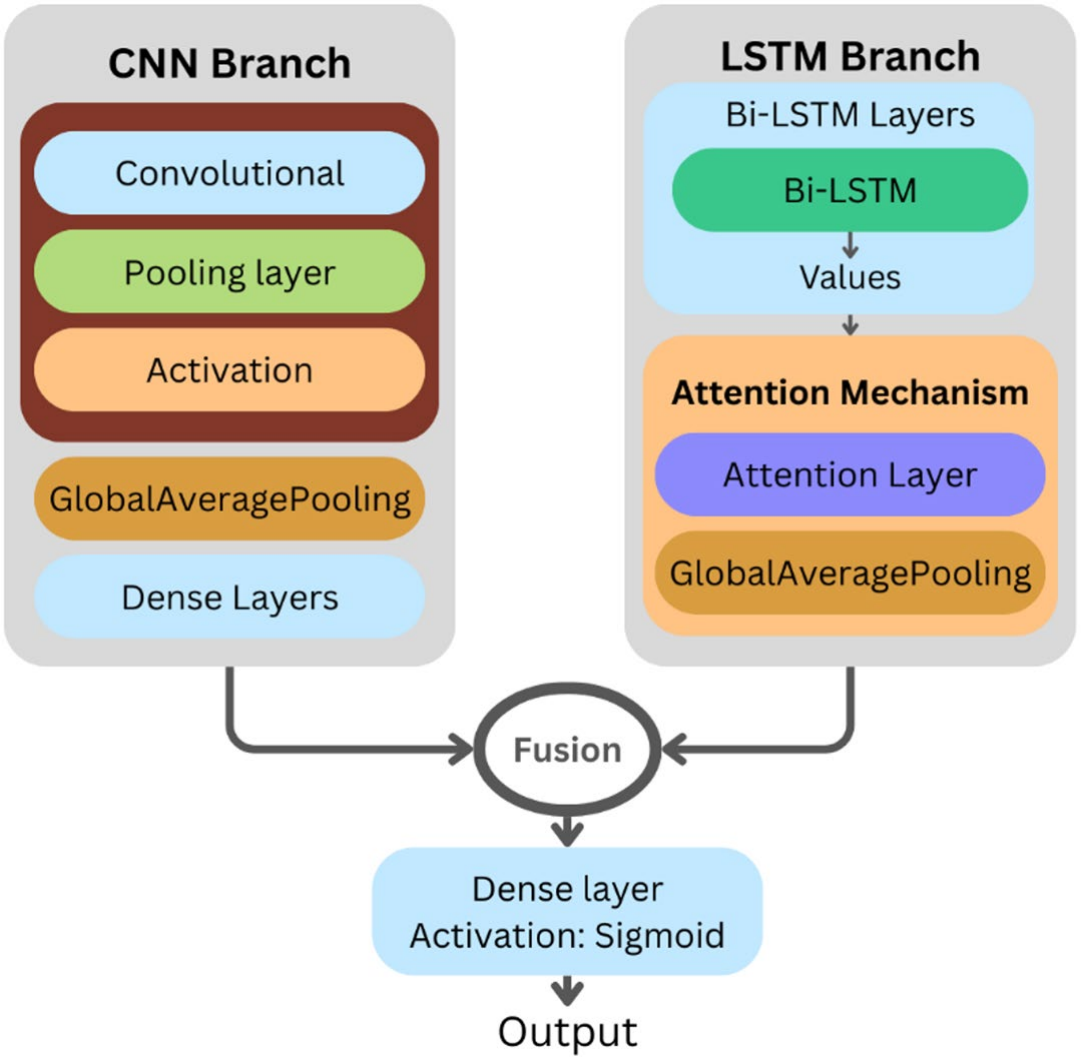
CNN-LSTM Architecture.

In this section we propose a hybrid CNN-LSTM with Attention Fusion architecture (Fig. 9) that combines a CNN-based feature extractor and an LSTM-based sequence processor with attention and fusion network. for a binary classification task. A sequence of shape (T, F), where T is the length of the sequence and F is the number of features, is given to the model.

CNN Branch: two successive 1D convolutional layers each followed by a max-pooling layer that process the input sequence. The first convolutional layer uses 256 size 5 filters with a ReLU activation, and 128 filters with size 3 are used in the second. Followed by a dense layer of size 128. The purpose of this structure is to extract a local feature hierarchy. A GlobalAveragePooling1D layer then processes the output, combining the features into a fixed-size vector.

LSTM Branch with Attention: the raw sequence input is passed through a Bidirectional LSTM of 256 units, configured to output the whole sequence of hidden states. This enables the model to capture context from previous and subsequent time steps. The Bi-LSTM output is then given to the self-attention layer, which computes a weighted average of the hidden states, with the weights calculated based on the relative importance of each time step. The output is a context vector, which is globally averaged.

Fusion and Classification: The feature vectors of the CNN and attention-enhanced LSTM branches are combined. The resulting representation is passed through a series of fully connected layers, including Dense and Dropout layers for regularization, and a normalization layer for training stabilization. The final layer is a single neuron with sigmoid activation function, giving the probability of the positive class for the binary classification problem.

**Figure:**
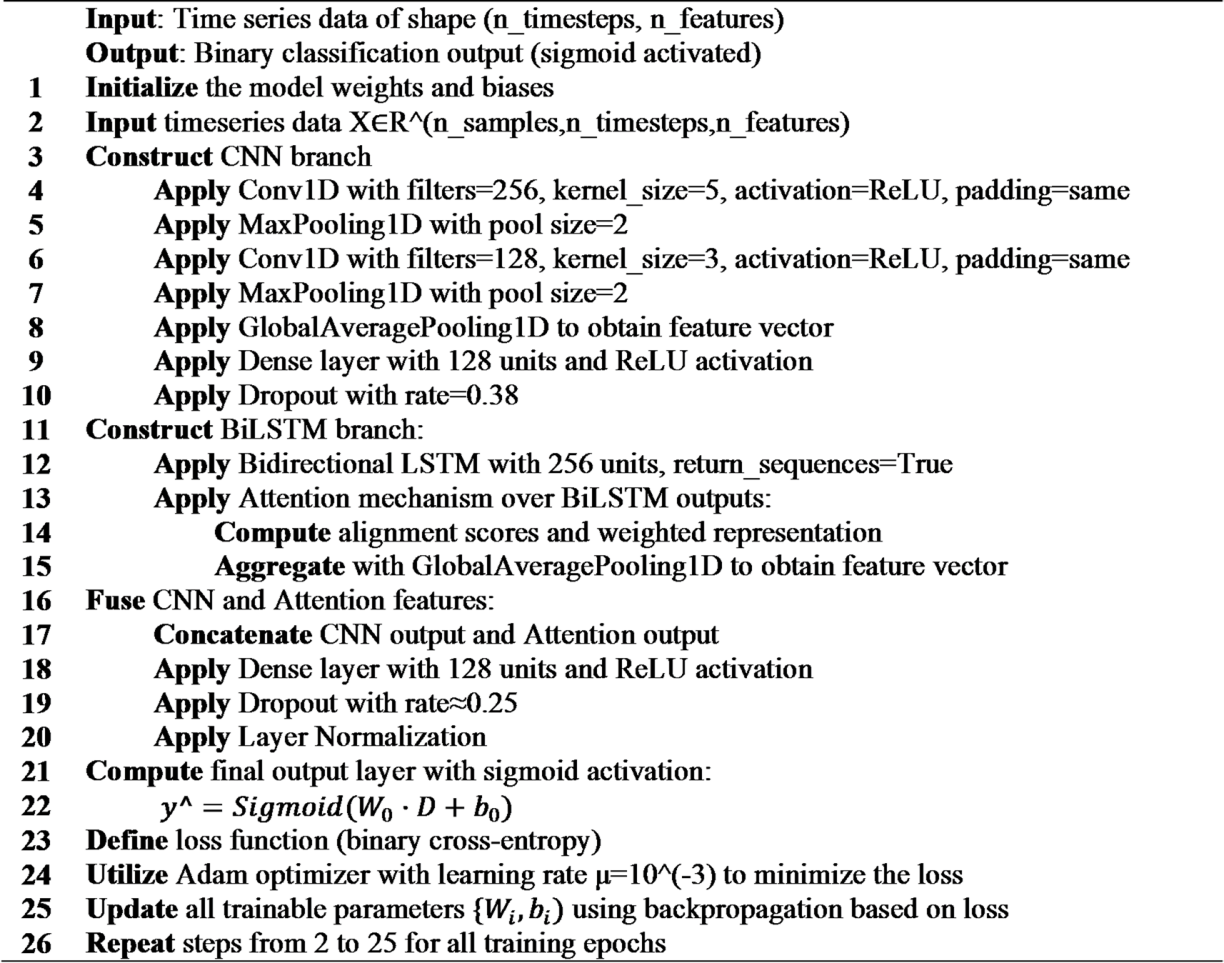
**Algorithm 9**: CNN–LSTM–Attention Fusion.

### Extended comparative evaluation

To ensure a comprehensive comparative evaluation of our hybrid CNN-LSTM with attention fusion model. We conducted another experiment implementing several other hybrid architectures that integrate CNN with recurrent based components, specifically CNN-LSTM, CNN-RNN, CNN-GRU, and CNN-BiLSTM. Similarly, for temporal convolutional models we assess hybridizations of TCN with the above RNN variants (TCN-LSTM, TCN-RNN, TCN-GRU, TCN-BiLSTM) to evaluate comparative performance in our target domain.

All the models were configured with the same settings and trained in the same conditions to ensure fairness. The individual models’ architectures are the same as the base models proposed in Sect.  3.3.

## Training configurations

### Loss function

To calculate the loss, we used binary cross-entropy loss (log loss) which is commonly used for binary classification problems for its theoretical and practical suitability^50^.

2 $$Binary cross-entropy= -[\boldsymbol{y}\cdot\:log({\boldsymbol{y}}^{\wedge})+(1-\boldsymbol{y})\cdot log(1-{\boldsymbol{y}}^{\wedge})]$$

Where:

- $\:y\in\:\left\{\mathrm{0,1}\right\}$ is the true label.
- ${\boldsymbol{y}}^{\wedge }\in\:\left(\mathrm{0,1}\right)$ is the predicted probability of class 1 (after sigmoid activation).

For a batch of size *N*, the average binary cross-entropy is

3 $$\:Loss=\:-\frac{1}{N}\:\sum\:_{i=1}^{N}{[y}_{i}\cdot\:\mathrm{log}\left({y}_{i}^{\wedge}\right)+(1-{y}_{i})\cdot\:\mathrm{l}\mathrm{o}\mathrm{g}(1-{y}_{i}^{\wedge})]$$

Where:

- $\:y\in\:\left\{\mathrm{0,1}\right\}$ is the true label.
- $\:{y}^{\wedge}\in\:\left(\mathrm{0,1}\right)$ is the predicted probability of class 1 (after sigmoid activation).
- $\:N$ is the total number of samples in the dataset.
- $\:i$ index of the current sample in the summation.

This loss function calculates the divergence between predicted probabilities and true labels. The binary cross-entropy penalizes confidence but wrong predictions more harshly and works best when output is a probability (passed through a sigmoid function) as in our models.

### Optimizer

To train the models we used Adam optimizer, which is widely known as one of the most effective optimization algorithms in deep learning^51^. Adam stands for Adaptive Moment Estimation, It combines two main ideas: momentum and RMSProp. Momentum gets the optimizer to remember the direction it was going in while RMSProp adjusts the learning rate for each parameter based on how often it gets updated.

Adam monitors the average of the squared gradients, which shows how much those mistakes vary, as well as the average of the gradients, which shows how much the model’s predictions are off. Adam will be able to change the model’s parameters more intelligently as a result.

Adam optimizer’s update rules are as follows:

- Computing the moving average of the gradients.

4 $$\:{m}_{t}={\beta\:}_{1}{m}_{t-1}+\left(1-{\beta\:}_{1}\right){g}_{t}$$

where:

- $\:{\beta\:}_{1}$ is the decay rate for gradient average.
- $\:{m}_{t-1}$ is the moving average of gradients from the previous time step t-1.
- $\:{g}_{t}$ is the gradient of the loss function at time step t.

b. Computing the moving average of the squared gradients.

5 $$\:{v}_{t}={\beta\:}_{2}{v}_{t-1}+\left(1-{\beta\:}_{2}\right){g}_{t}^{2}$$

where:

- $\:{v}_{t}$ is the moving average of squared gradients at timestep t.
- $\:{\beta\:}_{2}$ is the decay rate for moving average of squared gradients.
- $\:{v}_{t-1}$ is the moving average of squared gradients from the previous time step t-1.
- $\:{g}_{t}$ is the gradient at time step t.
- $\:{g}_{t}^{2}$ is the element-wise square of the gradient.

c. Correct the bias in these averages.

6 $$\:{\widehat{m}}_{t}=\frac{{m}_{t}}{1-{\beta\:}_{1}^{t}}$$

7 $$\:{\widehat{v}}_{t}=\frac{{v}_{t}}{1-{\beta\:}_{2}^{t}}$$

where:

- $\:{\widehat{m}}_{t}$ is the bias-corrected first moment estimate (corrected moving average of gradients).
- $\:{m}_{t}$ is the moving average of gradients at timestep t.
- $\:{\beta\:}_{1}$ is the decay rate for the first moment moving average.
- $\:{\widehat{v}}_{t}$ is the bias-corrected second moment estimate (corrected moving average of squared gradients).
- $\:{v}_{t}$ is the moving average of squared gradients at time step t.
- $\:{\beta\:}_{2}$ is the decay rate for the second moment moving average.

d. Update the parameters.

8 $$\:{\theta\:}_{t+1}={\theta\:}_{t}-\alpha\:\frac{\widehat{{m}_{t}}}{\sqrt{\widehat{{v}_{t}}}+\varepsilon}$$

where:

- $\:{\theta\:}_{t+1}$ is the updated model parameters at iteration t + 1.
- $\:{\theta\:}_{t}$ is the current model parameters at iteration t.
- $\:\alpha\:$ is the learning rate.
- $\:{\widehat{m}}_{t}$ is the bias-corrected first moment estimate.
- $\:{\widehat{v}}_{t}$ is the bias-corrected second moment estimate.
- $\:ϵ$ is a small constant added for numerical stability.

Adam has a distinct learning rate for each parameter, which allows the model to learn efficiently. It is faster and more precise than standard methods like traditional stochastic gradient descent (SGD).

### Hyperparameters optimization

Hyperband is a sophisticated hyperparameter optimization algorithm that effectively distributes computational resources by dynamically terminating underperforming configurations and allocating resources to promising configurations^52^. In contrast to grid search^53^, which thoroughly examines every potential parameter combination, or random search, which randomly picks configurations^54^.

Significant computational savings are achieved, and a more thorough investigation of the hyperparameter space within a set budget is made possible. In deep learning, where training each model can be resource-intensive, Hyperband’s adaptive allocation strategy works especially well since it allows for faster convergence to optimal or nearly ideal hyperparameter settings than conventional techniques^52^.

We implemented automated hyperparameter tuning using Keras Tuner’s Hyperband algorithm which dynamically allocates resources to promising configurations, optimizing for validation accuracy. Hyperparameters were optimized using Hyperband search with max 40 epochs per trial. The best model was selected based on validation accuracy. Early stopping was applied with a patience of 5 epochs.

### Preventing overfitting

To mitigate overfitting, the dataset was partitioned into training, validation, and test sets, with 15% of the data reserved for validation and 15% for testing. During hyperparameter optimization and model training, early stopping was employed: training was halted if the validation loss did not improve for five consecutive epochs. This approach ensures that model selection and tuning are based on generalization performance rather than training accuracy^55^. By monitoring validation metrics and limiting training duration for underperforming configurations, the risk of overfitting is minimized, resulting in a more robust and generalizable model^56^.

## Results

### Computational environment

The experiment was conducted through Kaggle environment, which provides a P100 GPU processor with 16GB VRAM, 29 GB RAM, and 57 GB disk space.

### Computational time

The time spent on data preprocessing, which involved standardization, feature extraction, handling missing values, and segmentation for the three farms, was approximately 40 min on a computing environment with a P100 GPU processor with 16 GB VRAM and 29 GB RAM. The model training time varied by the complexity of the models and the number of epochs required for convergence. Generally, more complex models needed more time per epoch and, in some cases, more epochs overall.

Base models computational efficiency was highest for the 1D-CNN and SimpleRNN models, with fewer parameters and thus experienced faster training times of around 5 to 12 min on the dataset. The TCN and Bi-LSTM models were less efficient because they used more parameters and would thus require a greater time to complete the model training of around 12 to 20 for these models. They capture long-range dependencies effectively at double the computational cost. The most computationally demanding model was the hybrid CNN-LSTM-Attention; while powerful, it was around 4× slower than the 1D-CNN, suitable only when performance justifies the cost. GPU acceleration was critical for managing these workloads, reducing computational time for the large dataset and complex architectures.

### Performance metrics

Performance metrics are necessary to verify the effectiveness of the models. The model has been evaluated on the following performance metrics: Accuracy, Precision, Recall, F1-Score, Area Under the Curve, and False Alarm Rate (FAR)^57^.

In order to assess the performance of the anomaly detection several metrics were employed to quantify its robustness and reliability in the detection of wind turbine faults. These metrics rely on the confusion matrix that categorizes the predictions into true positives (TP: correct anomalies), true negatives (TN: correct normal conditions), false positives (FP: normal conditions misclassified as anomalies), and false negatives (FN: missed anomalies). The following metrics were computed using Eqs. (9–13). While these measures are standard in the machine learning domain, their interpretation carries direct operational relevance in wind turbine fault detection. Specifically:

- Accuracy.

Accuracy measures the proportion of correctly classified samples for normal and anomaly classes. While helpful to estimate overall performance, it is misleading in datasets with imbalances^58^:

9 $$\:Accuracy=\frac{TP+TN}{TP+TN+FP+FN}$$

- Precision.

Precision evaluates the model’s ability to avoid false alarms, which is most crucial for minimizing unnecessary maintenance interventions and reducing operational costs in wind farm operations^59^:

10 $$\:Precision=\frac{TP}{TP+FP}$$

- Recall (Sensitivity).

Recall evaluates the model’s ability to detect true anomalies, which matters most in preventing catastrophic failures. This metric is critical in wind turbines because missed faults (false negatives) can result in costly downtime, reduced energy production, or equipment damage^59^:

11 $$\:Recall=\frac{TP}{TP+FN}$$

- F1-Score.

The F1-Score trades off precision and recall into a single score, providing an unbiased assessment of model performance in class-imbalanced settings^60^. particularly valuable when both missed faults and false alarms carry significant costs:

12 $$\:F1-Score=\frac{2\times\:Precision\times\:Recall}{Precision+Recall}$$

- Area Under the Curve (AUC).

AUC measures the model’s ability to discriminate between normal and faulty conditions across different thresholds of the decision boundary. A higher AUC indicates a more robust model, as it reflects strong detection capability regardless of where the classification threshold is set.

- False Alarm Rate (FAR).

FAR measures the quantity of false anomaly alerts, which directly affect operational costs and system trust^61^:

13 $$\:FAR=\frac{FP}{TN+FP}$$

### Experiments

**Fig. 10:**
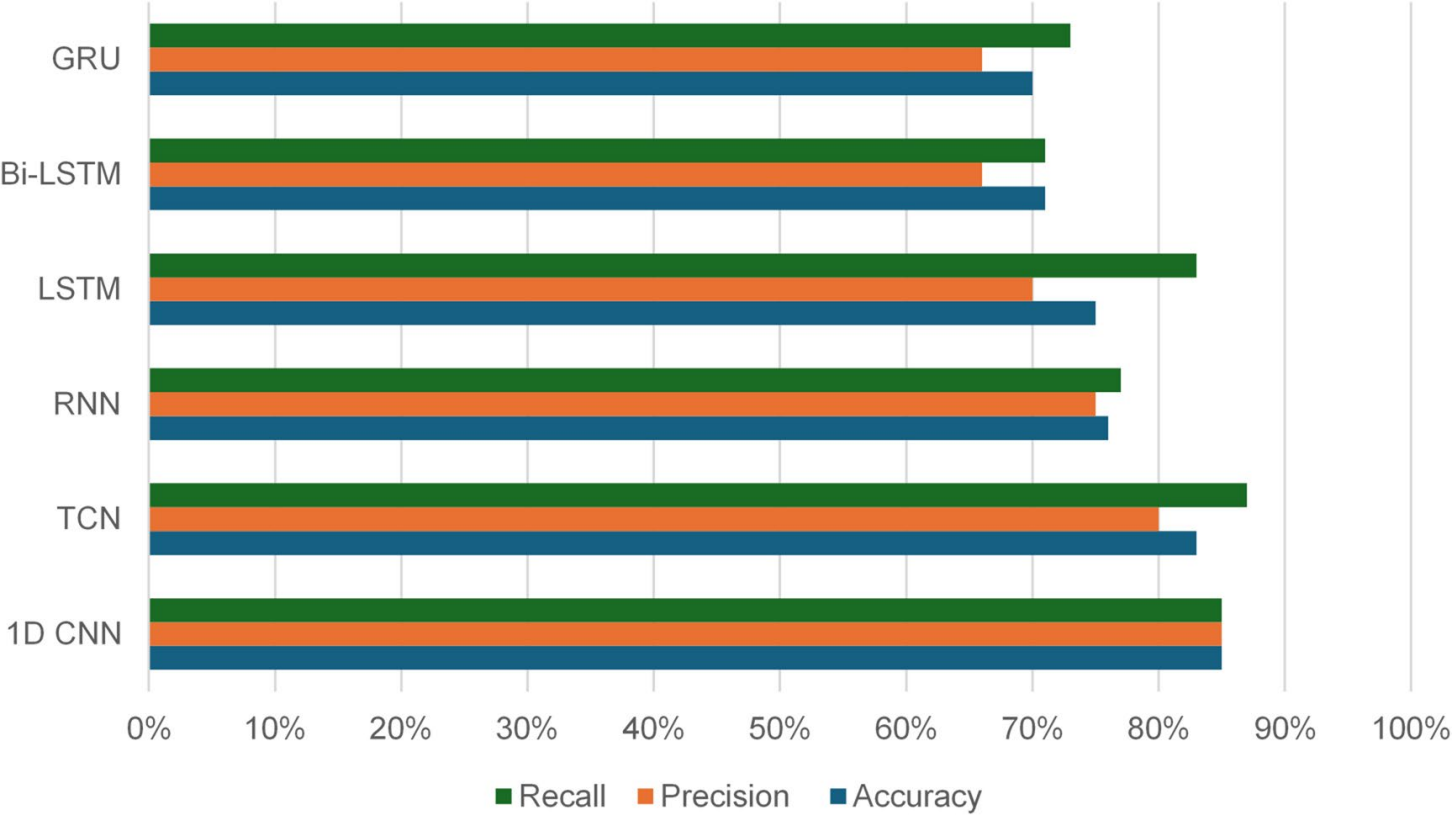
Evaluation results.

**Fig. 11:**
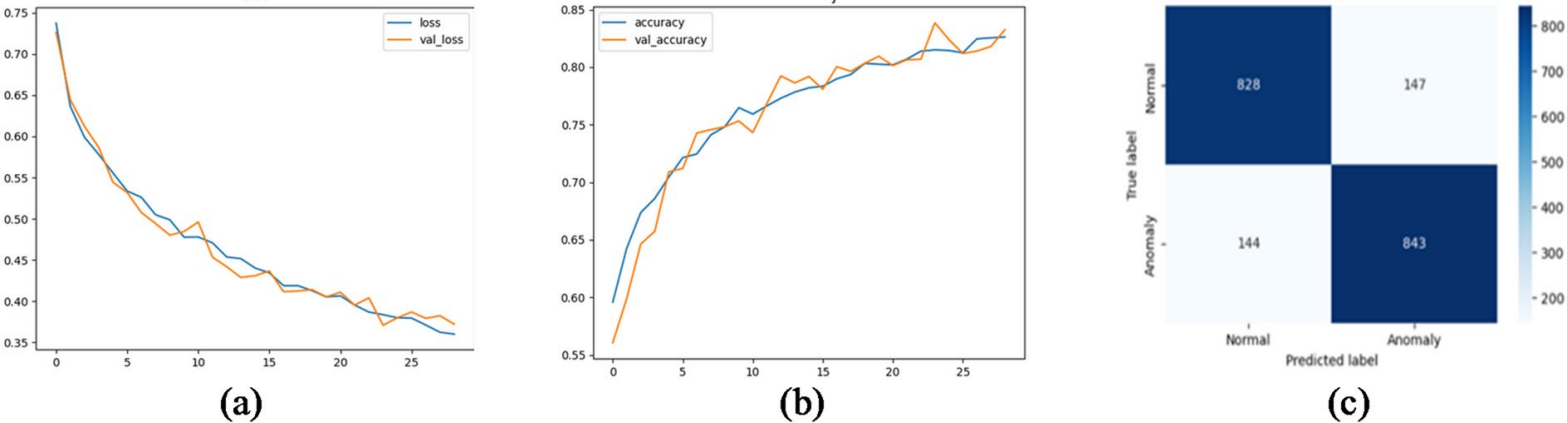
1D-CNN training curve and confusion matrix; (**a**) loss curve; (**b**) accuracy curve; (**c**) confusion matrix.

**Fig. 12:**
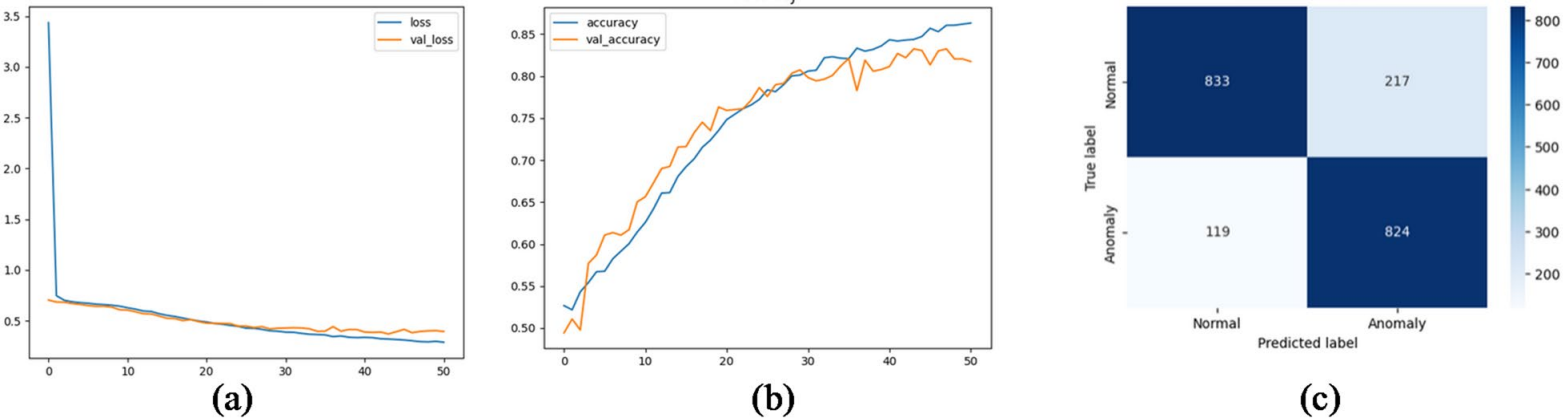
TCN training curve and confusion matrix (**a**) loss curve; (**b**) accuracy curve; (**c**) confusion matrix.

**Fig. 13:**
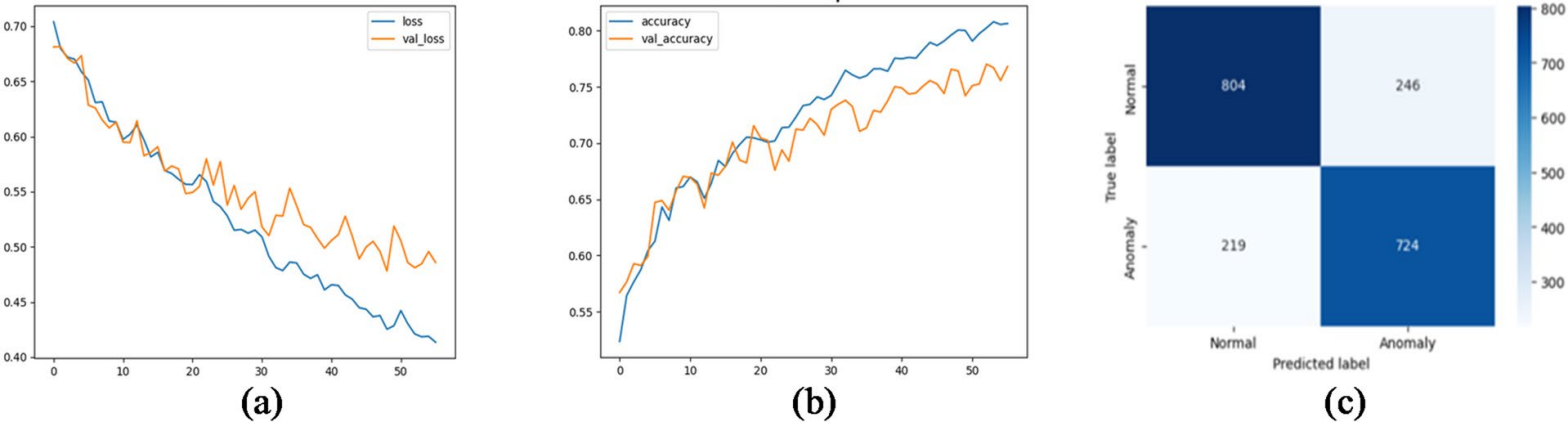
RNN training curve and confusion matrix (**a**) loss curve; (**b**) accuracy curve; (**c**) confusion matrix.

**Fig. 14:**
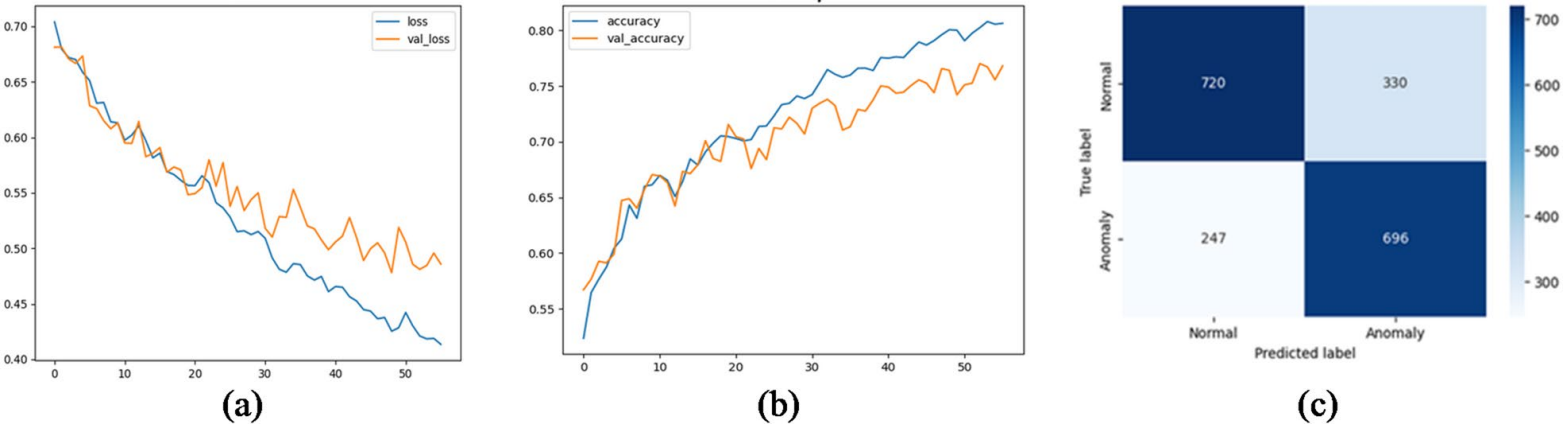
LSTM training curve and confusion matrix (**a**) loss curve; (**b**) accuracy curve; (**c**) confusion matrix.

**Fig. 15:**
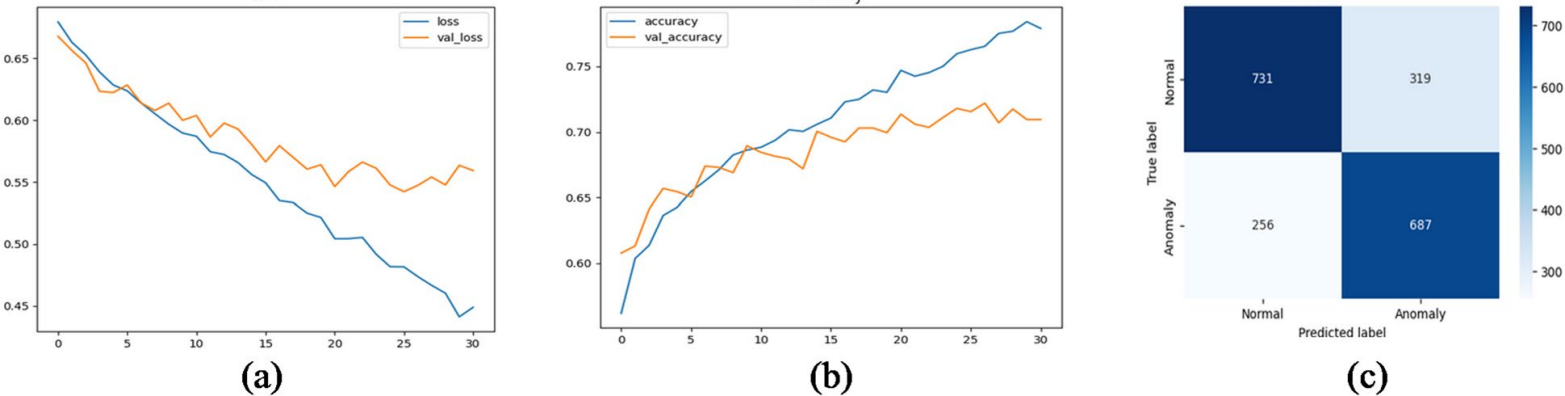
Bi-LSTM training curve and confusion matrix (**a**) loss curve; (**b**) accuracy curve; (**c**) confusion matrix.

**Fig. 16:**
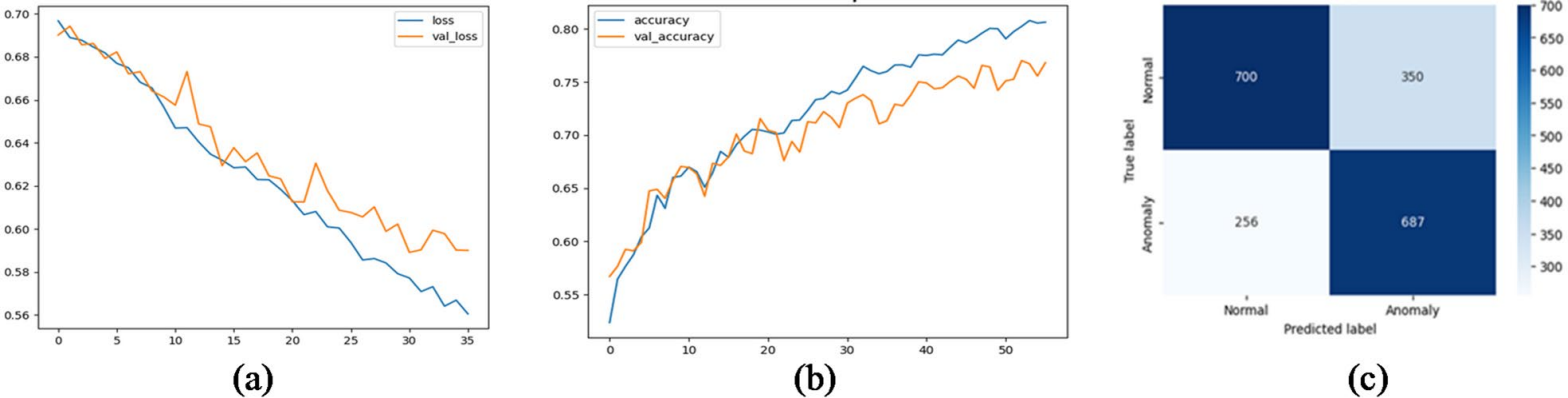
GRU training curve and confusion matrix (**a**) loss curve; (**b**) accuracy curve; (**c**) confusion matrix.

We evaluated several deep learning architectures on the processed time-series SCADA wind turbines dataset comparing their classification performance using accuracy, precision, recall, false alarm rate and F1-score. Feature engineering with temporal attributes (hour, day, month) further enhanced model performance. The training curves shows stable convergence with minimal overfitting, due to the use of dropout and L2 regularization. Table 10; Fig. 10 show the evaluation matrices of the models. Figures 11, 12, 13, 14, 15 and 16 present the learning curves and confusion matrix for each model.

**Table 10:** Models evaluation.

Model	Accuracy	Precision	Recall	F1	AUC	FAR
1D CNN	**85%**	**85%**	**85%**	**85%**	**94%**	**15%**
TCN	83%	80%	87%	83%	89%	20%
RNN	76%	75%	77%	76%	86%	23%
LSTM	75%	70%	83%	76%	84%	30%
Bi-LSTM	71%	66%	71%	73%	80%	30%
GRU	70%	66%	73%	69%	76%	33%

### Model performance on individual datasets

In our dataset, wind farm C clearly contributes more data samples than wind farms A and B. Specifically, Wind Farm C contributes approximately 61% of the total dataset, while Wind Farms A and B contribute 23% and 16%, respectively. This could result in the model being biased toward wind farm C data distribution. To analyze the impact of each wind farm’s data distribution on the training outcomes, we have tested the model individually on each test data of all the wind farms. The results indicate that while all farms performance is comparable, wind farm C does in fact perform slightly better than the other two farms. This imbalance raises the possibility of model bias toward the dominant dataset.

While in this study we chose direct merging of datasets, approaches such as data balancing or weighted loss functions could be used to address farm-level imbalance. We discuss this as consideration for future extensions.

To address concerns regarding the impact of temporal discrepancies on model performance, we conducted additional experiments training and evaluating our model separately on each individual dataset. Individual analysis results were then contrasted with results from testing the model when trained on the combined dataset across all years. Table 11 displays the results for each wind farm and for the combined dataset.

**Table 11:** Individual farms models accuracy.

Feature	Farm A	Farm B	Farm C	Combined
Accuracy	83%	91%	81%	85%

Our results show the models trained on single datasets performed competitively, but the combined dataset model demonstrated higher accuracy than Wind farm A and C models, while Wind Farm B performed petter than the combined dataset model. We believe this is due to the very small number of wind turbines and events on Wind Farm B which make the model familiar with the events and patterns included in Wind Farm B but would struggle to generalize to data from other sources. This shows that using data from multi-year datasets does not deteriorate and might even enhance the model’s generalization to different temporal conditions.

### Statistical significance

We conducted paired t-tests for the 1D-CNN with each baseline model across five runs to validate that the observed performance gains of the 1D-CNN were not due to random variation. This analysis allows us to determine whether the differences in model performance are statistically significant rather than coincidental.

The results (Table 12) show that the 1D-CNN is consistently superior to any of the baseline models with statistically significant differences (*p* < 0.05) in almost every case. The difference is very strong in relation to recurrent architectures like LSTM, Bi-LSTM, and GRU which show p-values below 0.001. The only case in which we got a borderline result (*p* = 0.05) was with TCN indicating that while we still observe evidence of 1D-CNN being a better performer; the advantage over TCN is less pronounced than with the other models.

**Table 12:** paired t-test results compared to 1D-CNN.

Model	t-value	*p*-value
TCN	2.71	0.05
RNN	6.18	0.0035
LSTM	9.52	0.0007
Bi-LSTM	17.14	0.0001
GRU	24.3	0.0001

Overall, these results provide statistical evidence that the observed differences in performance from using 1D-CNN are unlikely to be a result of random noise. This improves the confidence in our conclusion that 1D-CNN would be a better architecture choice for fault detection in wind turbines under the study conditions.

### Decision making

For the selection of the most suitable model for our task, we considered multiple performance metrics, including accuracy, precision, recall, false alarm rate, and F1-score, alongside training efficiency. In our problem we’ve taken all matrices into consideration and prioritized accuracy and precision. While all the models were able to learn from datasets, the 1D-CNN architecture consistently achieved the highest accuracy and balanced precision and recall, indicating its higher ability to capture temporal dependencies.

### Hybrid CNN-LSTM with attention fusion

The hybrid CNN-LSTM Attention model was trained and tested on the same proposed wind turbine fault detection dataset. The model was set to train for 100 epochs while monitoring the value of validation loss for early stopping. Model performance was tracked using binary cross-entropy loss and accuracy metrics on both the training and validation datasets. The model was successfully learning from the data, as evidenced by the training loss’s steady decline over the epochs. Although there were a few small fluctuations, the validation loss also displayed a similar declining trend, demonstrating the model’s capacity for generalization.

**Fig. 17:**
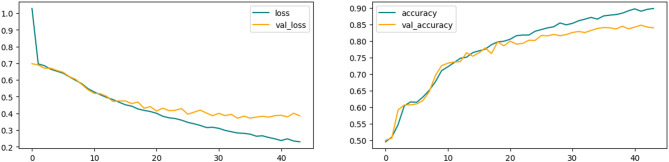
CNN-LSTM attention fusion training curves.

The model’s performance on the validation set shows that it was able to learn to generalize outside of the training set. It scored 87% accuracy on the test set, and the same score on precision, and recall, detailed evaluation results used in Table 13. The results show that the suggested hybrid CNN-LSTM Attention model is a successful sequence classification architecture. With the help of a strong attention mechanism, it can learn both long-range temporal dependencies and local features simultaneously, which enables it to perform well in generalization and achieve high accuracy. Figure 17 shows the accuracy and loss curves of the model training.

**Table 13:** CNN-LSTM attention fusion evaluation.

Model	Accuracy	Precision	Recall	F1	AUC	FAR
CNN-LSTM attention fusion	87%	87%	87%	87%	95%	12%

### Extended comparative evaluation

The comparative study involved the hybrid models detailed in Sect.  3.5, where CNN- and TCN-based hybrid architectures were combined with different recurrent units to evaluate performance under consistent training conditions. This section presents the results of the comparative evaluation of hybrid deep learning models for the classification of multivariate time series faults in wind turbines. The assessed models are hybrids of 1D-CNN and TCN, incorporating different recurrent architectures, including LSTM, vanilla RNN, GRU, and Bidirectional LSTM. The result of this experiment shown in Table 14 (CNN Based Hybrid Models) and Table 15 (TCN Based Hybrid Models), demonstrates that the CNN-LSTM Attention fusion model outperforms both traditional and hybrid architectures.

**Table 14:** Performance comparison of 1D-CNN based hybrid models.

Model	CNN-LSTM	CNN-BiLSTM	CNN-RNN	CNN-GRU	Proposed CNN-LSTM-attention
Accuracy	86%	80%	83%	81%	87%

**Table 15:** Performance comparison of TCN based hybrid models.

Model	TCN-LSTM	TCN-BiLSTM	TCN-RNN	TCN-GRU	Proposed CNN-LSTM-Attention
Accuracy	82%	79%	80%	79%	87%

## Conclusion and future work

The use of deep learning architectures to the classification of time series data from a novel dataset. which was produced by merging data from three different sources—is thoroughly examined in this research. Using a SCADA time-series dataset, we investigated the efficacy of various deep learning architectures for time-series classification in this study, including CNN, TCN, RNN, LSTM, Bi-LSTM, and GRU. In order to identify underlying patterns, the preprocessing involved meticulous normalization, feature selection, and temporal feature engineering. The findings of the experiment demonstrated that while all evaluated models could learn from the data, the 1D-CNN outperformed the others and produced the best performance matrices.

Our results show that a well-structured 1D-CNN architecture provides a very effective and efficient solution for the problem when paired with strong preprocessing and feature engineering. This demonstrates how convolutional layers, which are backed by computational efficiency, are successful in identifying local temporal patterns and features in complicated, heterogeneous time series data.

The results also confirm that careful preprocessing of heterogeneous datasets is essential to model performance and could improve generalizability. This aligns with findings by Chen et al.^47^, who highlighted the importance of standardized resampling and normalization when integrating energy data originating from multiple sources. Approaches like data balancing or weighted loss functions can effectively mitigate farm-level imbalances, and we highlight these strategies as important considerations for future work. Building on these insights, our study provides a benchmarking framework for evaluating deep learning models under consistent preprocessing conditions.

Our findings highlight the significance of integrating several datasets, thorough preparation, and intricate deep architecture design in resolving difficult time series classification issues. This work emphasizes the relative performance of commonly used baseline deep learning models across heterogeneous wind farm datasets. This benchmarking framework provides a strong starting point from which future work can evaluate and compare architectural variations.

Building on this foundation, we put forward and verified a new CNN-LSTM hybrid model that incorporates an attention mechanism. This architectural enhancement performed better, which we investigated in a follow-up experiment. The attention mechanism enables the hybrid model to dynamically focus on the most useful characteristics for the classification task, while the convolutional and recurrent layers’ complementary strengths are effectively utilized.

This study confirms the efficacy of sophisticated hybrid architectures and offers a solid, repeatable foundation for wind turbine fault detection. Our findings could now be expanded upon in future research by investigating additional architectural enhancements, like residual learning strategies, or by utilizing Explainable AI (XAI) techniques to delve deeper into model interpretability and prediction accuracy.

## Data Availability

The data that support the findings of this study are available within this article.
